# Engineered exosomes as an in situ DC-primed vaccine to boost antitumor immunity in breast cancer

**DOI:** 10.1186/s12943-022-01515-x

**Published:** 2022-02-11

**Authors:** Lanxiang Huang, Yuan Rong, Xuan Tang, Kezhen Yi, Peng Qi, Jinxuan Hou, Weihuang Liu, Yuan He, Xing Gao, Chunhui Yuan, Fubing Wang

**Affiliations:** 1 Department of Laboratory Medicine, Zhongnan Hospital of Wuhan University, No.169 Donghu Road, Wuhan, Wuchang District, China; 2 Center for Single-Cell Omics and Tumor Liquid Biopsy, Zhongnan Hospital of Wuhan University, Wuhan, China; 3 Department of Thyroid and Breast Surgery, Hubei No. 3 People’s Hospital of Jianghan University, Wuhan, China; 4 Department of Thyroid and Breast Surgery, Zhongnan Hospital of Wuhan University, Wuhan, China; 5 Medical Research Center for Structural Biology, School of Basic Medical Sciences, Wuhan University, Wuhan, China; 6 Animal Experiment Center, Zhongnan Hospital of Wuhan University, Wuhan, China; 7 Department of Laboratory Medicine, Wuhan Children’s Hospital, Tongji Medical College, Huazhong University of Science and Technology, Wuhan, China; 8 Wuhan Research Center for Infectious Diseases and Cancer, Chinese Academy of Medical Sciences, Wuhan, China

**Keywords:** Breast cancer, Tumor-derived exosomes, Immunogenic cell death, Hiltonol, Type 1 conventional dendritic cells, DC vaccine

## Abstract

**Background:**

Dendritic cells (DCs) are central for the initiation and regulation of innate and adaptive immunity in the tumor microenvironment. As such, many kinds of DC-targeted vaccines have been developed to improve cancer immunotherapy in numerous clinical trials. Targeted delivery of antigens and adjuvants to DCs in vivo represents an important approach for the development of DC vaccines. However, nonspecific activation of systemic DCs and the preparation of optimal immunodominant tumor antigens still represent major challenges.

**Methods:**

We loaded the immunogenic cell death (ICD) inducers human neutrophil elastase (ELANE) and Hiltonol (TLR3 agonist) into α-lactalbumin (α-LA)-engineered breast cancer-derived exosomes to form an in situ DC vaccine (HELA-Exos). HELA-Exos were identified by transmission electron microscopy, nanoscale flow cytometry, and Western blot analysis. The targeting, killing, and immune activation effects of HELA-Exos were evaluated in vitro. The tumor suppressor and immune-activating effects of HELA-Exos were explored in immunocompetent mice and patient-derived organoids.

**Results:**

HELA-Exos possessed a profound ability to specifically induce ICD in breast cancer cells. Adequate exposure to tumor antigens and Hiltonol following HELA-Exo-induced ICD of cancer cells activated type one conventional DCs (cDC1s) in situ and cross-primed tumor-reactive CD8^+^ T cell responses, leading to potent tumor inhibition in a poorly immunogenic triple negative breast cancer (TNBC) mouse xenograft model and patient-derived tumor organoids.

**Conclusions:**

HELA-Exos exhibit potent antitumor activity in both a mouse model and human breast cancer organoids by promoting the activation of cDC1s in situ and thus improving the subsequent tumor-reactive CD8^+^ T cell responses. The strategy proposed here is promising for generating an in situ DC-primed vaccine and can be extended to various types of cancers.

**Graphic Abstract:**

Scheme 1. Schematic illustration of HELA-Exos as an in situ DC-primed vaccine for breast cancer. (A) Allogenic breast cancer-derived exosomes isolated from MDA-MB-231 cells were genetically engineered to overexpress α-LA and simultaneously loaded with the ICD inducers ELANE and Hiltonol (TLR3 agonist) to generate HELA-Exos. (B) Mechanism by which HELA-Exos activate DCs in situ in a mouse xenograft model ofTNBC. HELA-Exos specifically homed to the TME and induced ICD in cancer cells, which resulted in the increased release of tumor antigens, Hiltonol, and DAMPs, as well as the uptake of dying tumor cells by cDC1s. The activated cDC1s then cross-primed tumor-reactive CD8+ T cell responses. (C) HELA-Exos activated DCs in situ in the breast cancer patient PBMC-autologous tumor organoid coculture system. Abbreviations: DCs: dendritic cells; α-LA: α-lactalbumin; HELA-Exos: Hiltonol-ELANE-α-LA-engineered exosomes; ICD: immunogenic cell death; ELANE: human neutrophil elastase; TLR3: Toll-like receptor 3; TNBC: triple-negative breast cancer; TME: tumor microenvironment; DAMPs: damage-associated molecular patterns; cDC1s: type 1 conventional dendritic cells; PBMCs: peripheral blood mononuclear cells

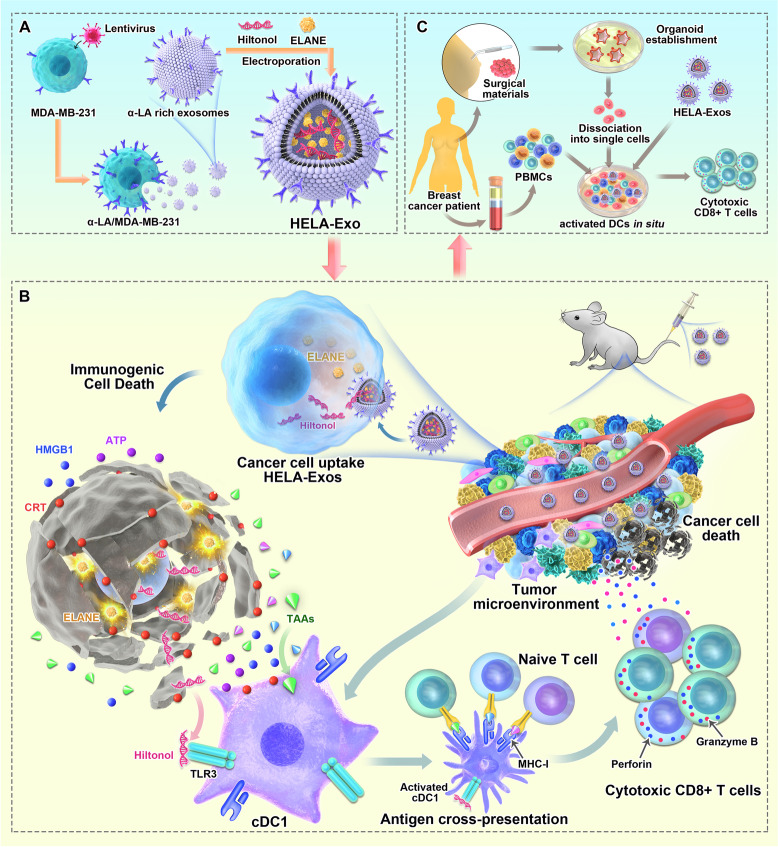

**Supplementary Information:**

The online version contains supplementary material available at 10.1186/s12943-022-01515-x.

## Introduction

As the most potent antigen-presenting cells (APCs), dendritic cells (DCs) are central to the initiation and regulation of innate and adaptive immunity in the tumor microenvironment (TME), with the ability to present tumor-associated antigens (TAAs) on MHC molecules and provide costimulatory/soluble factors to shape antitumor T cell responses [1]. As such, many kinds of DC-targeted vaccines have been developed to improve cancer immunotherapy in numerous clinical trials [2, 3]. Targeted delivery of antigens and adjuvants to DCs *in vivo* represents an important approach to enhance the antitumor effects of DCs [4]. In contrast to ex vivo generation, in vivo activation and mobilization of DCs allows vaccines to be produced on a larger scale and allows direct activation of natural DC subsets at multiple sites in vivo [5]. However, because DC-target receptors, such as DEC205 [6] and DC-SIGN [7], are not exclusively expressed on tumor-infiltrating DCs, this strategy can also induce antigen-nonspecific activation of the immune system and potential side effects in the clinic [8, 9]. In addition, the other major challenge for the implementation this strategy is the identification of optimal immunodominant tumor antigens, such as TAA, TSA and even patient-specific neoantigens [10]. Efforts to address these concerns are critical for making DC vaccines clinically feasible.

The type of cell death induced in tumor cells can influence their efficacy in inducing immunity [11]. Immunogenic cell death (ICD) enhances tumor antigen exposure, boosts the release of immune-stimulating tumor cell content, and favors the uptake of dying tumor cells by DCs [12–14]. In this context, ICD has been approved for whole-tumor lysate preparations for autologous DC vaccines [15]. Thus, induction of ICD in tumor cells would be an effective way to maintain adequate exposure of DCs to tumor antigens and bypass the need for identification of optimal immunodominant antigens. On the other hand, DCs usually exhibit poor maturation in the TME and are thus less effective in presenting tumor antigens [16]. Adjuvants that drive immunogenic DC activation, particularly agonists of TLR3, are being actively investigated [6]. Administration of TLR3 agonists is extraordinarily effective in inducing maturation of type one conventional DCs (cDC1s) [17, 18], which are necessary for inducing cellular immunity and are linked to increased survival in patients with certain cancer types due to their efficient processing and cross-presentation of exogenous antigens on MHC class I molecules to activate CD8 ^+^ T cells and prime T_H_1 cell responses [19–22]. Thus, targeted delivery of ICD inducers and TLR3 agonists into tumor cells would be an ideal way to activate tumor-infiltrating DCs in situ and avoid interactions in the immune milieu of other sites.

In recent years, exosomes have come into focus as “natural nanoparticles” for use as drug delivery vehicles owing to their low cytotoxicity, ability to maximize the bioavailability of drugs, and exquisite target-homing specificity [23, 24]. The therapeutic potential of exosomes is reflected by their application in an increasing number of clinical trials (ClinicalTrials.gov) for the treatment of chronic kidney diseases [25], non-small-cell lung cancer [26], colon cancer [27], type 1 diabetes, and severe COVID-19 [28]. Notably, the chemotherapeutic doxorubicin (Dox) was more stable when loaded in breast cancer-derived exosomes and accumulated more efficiently in tumors than free Dox for the treatment of breast cancer in a mouse model [29]. Therefore, tumor-derived exosomes (Texs) can be used as a cell-free therapeutic carrier to infiltrate the TME for in situ DC activation.

**Scheme 1:**
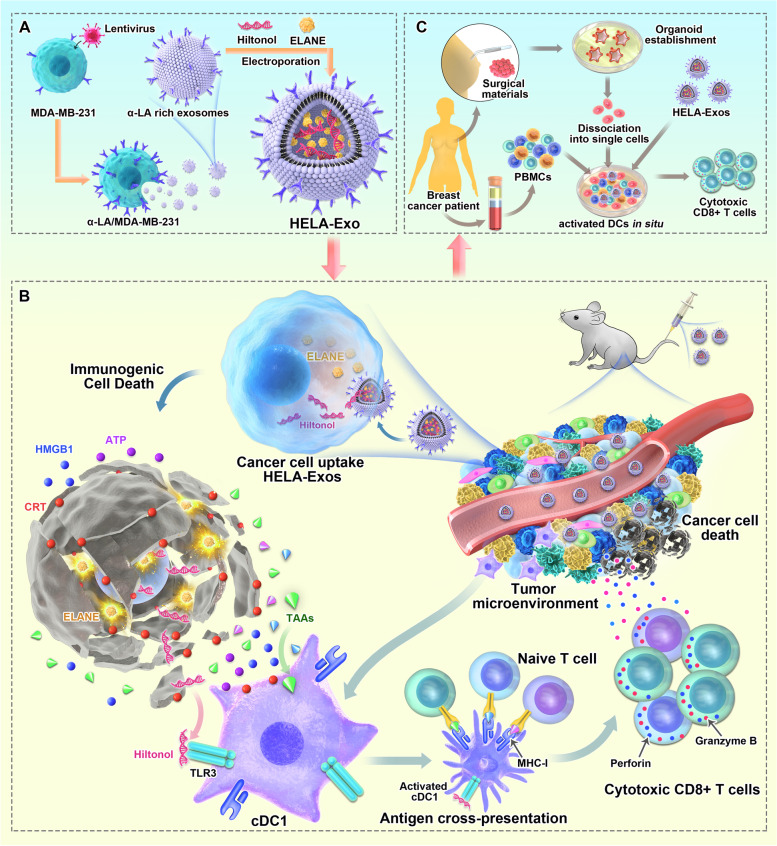
Schematic illustration of HELA-Exos as an *in situ* DC-primed vaccine for breast cancer. **A** Allogenic breast cancer-derived exosomes isolated from MDA-MB-231 cells were genetically engineered to overexpress α-LA and simultaneously loaded with the ICD inducers ELANE and Hiltonol (TLR3 agonist) to generate HELA-Exos. **B** Mechanism by which HELA-Exos activate DCs *in situ *in a mouse xenograft model ofTNBC. HELA-Exos specifically homed to the TME and induced ICD in cancer cells, which resulted in the increased release of tumor antigens, Hiltonol, and DAMPs, as well as the uptake of dying tumor cells by cDC1s. The activated cDC1s then cross-primed tumor-reactive CD8^+^ T cell responses. (**C**) HELA-Exos activated DCs *in situ *in the breast cancer patient PBMC-autologous tumor organoid coculture system. Abbreviations: DCs: dendritic cells; α-LA: α-lactalbumin; HELA-Exos: Hiltonol-ELANE-α-LA-engineered exosomes; ICD: immunogenic cell death; ELANE: human neutrophil elastase; TLR3: Toll-like receptor 3; TNBC: triple-negative breast cancer; TME: tumor microenvironment; DAMPs: damage-associated molecular patterns; cDC1s: type 1 conventional dendritic cells; PBMCs: peripheral blood mononuclear cells

Herein, we developed and optimized a formulation of target-specific exosomes loaded with Hiltonol (TLR3 agonist) and the ICD inducer human neutrophil elastase (ELANE) [30] to form an in situ DC vaccine for breast cancer treatment. α-Lactalbumin (α-LA), a breast-specific immunodominant protein expressed in the majority of human breast cancers [31], was further genetically enriched on the surface of exosomes as a specific tumor-homing protein to enhance targeting capability and immunogenicity [32]. In both a mouse model and patient-derived tumor organoids, these Hiltonol-ELANE-α-LA-engineered exosomes (HELA-Exos) possessed a profound ability to accumulate in cancer cells after systemic administration and exerted improved therapeutic effects compared to those of free Hiltonol (Scheme 1). Therefore, the combination of TLR3 agonists with ICD inducers based on cell-free exosomes offers a powerful and novel therapeutic platform for designing DC vaccines for breast cancer.

## Results

### Characterization and optimization of HELA-Exos

**Fig. 1:**
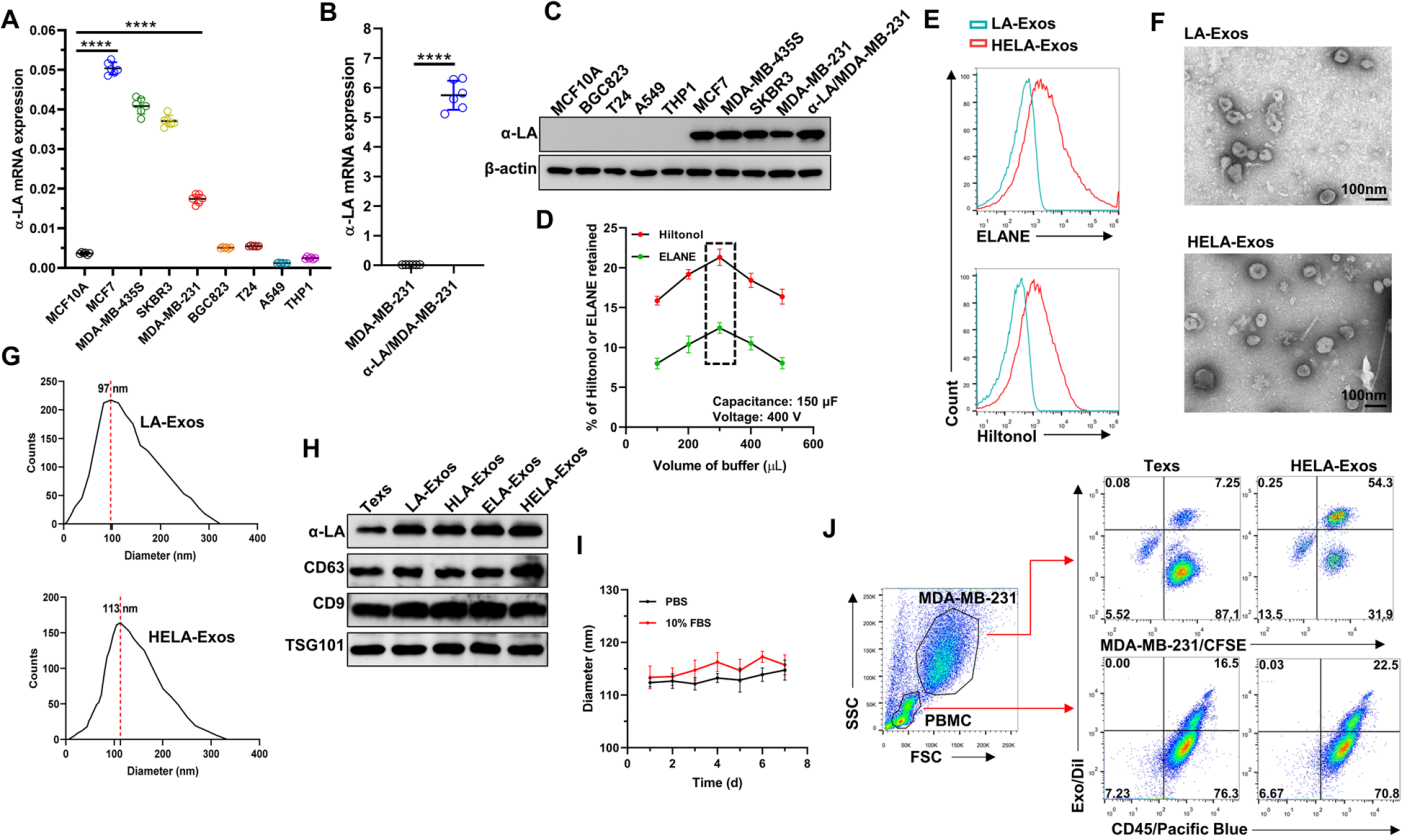
Preparation, characterization, and targeting of HELA-Exos. **A and B** Relative abundance of α-LA mRNA in different cell lines (the human normal breast epithelial cell line MCF-10A, breast adenocarcinoma cell line MCF7, breast cancer cell line MDA-MB-435S, breast cancer cell line SKBR3, TNBC cell line MDA-MB-231, gastric adenocarcinoma cell line BGC-823, transitional cell carcinoma cell line T24, alveolar basal epithelial adenocarcinoma cell line A549, and leukemic cell line THP-1) and infected MDA-MB-231 cells by qPCR; data were normalized to GAPDH. The data are presented as the mean ± SD; *n* = 6. A *t test* was performed for statistical analysis (****: *P* < 0.0001). **C** Western blot analysis for detecting the expression of α-LA protein in cells as in **A**. β-Actin was used as a loading control. **D** FITC-labeled ELANE and CX-Rhodamine-labeled Hiltonol were electroporated with exosomes at 400 V 150 µF in the volume of buffer shown on the x-axis, and ELANE and Hiltonol loading were determined based on the fluorescent standard curve. The data are presented as the mean ± SD; *n* = 3. **E** The loading of ELANE and Hiltonol in exosomes was characterized by nanoscale flow cytometry. **F** Transmission electron micrograph of LA-Exos and HELA-Exos. Scale bar, 100 nm. **G** Size distribution of LA-Exos and HELA-Exos measured by a particle sizer. **H** Western blot analysis for detecting the expression of the α-LA protein and exosomal markers in Texs, LA-Exos, HLA-Exos, ELA-Exos, and HELA-Exos. **I** Diameter change of HELA-Exos from day 1 to day 7. The data are presented as the mean ± SD; *n* = 3. **J** Flow cytometry analysis of the cellular uptake of MDA-MB-231 cells or PBMCs after incubation with Texs-DiI or HELA-Exos-DiI for 2 h in the coculture model

To determine whether α-LA is a specific dominant epitope in breast cancer, we first evaluated the expression of α-LA in different cell lines. As predicted, the triple-negative breast cancer (TNBC) cell line MDA-MB-231 and the non-TNBC cell lines MCF7, MDA-MB-435S, and SKBR3 expressed significantly higher α-LA mRNA levels than other cell lines, including the nontransformed human mammary epithelial cell line MCF10A and other types of cancer cells (Fig. 1A). TNBC represents a class of poorly immunogenic tumors associated with low survival rates and high resistance to current immunotherapies [33, 34]. Therefore, MDA-MB-231 cells were genetically engineered to overexpress α-LA (Fig. 1B and C), and exosomes derived from MDA-MB-231 cells (Texs) and α-LA-overexpressed cells (LA-Exos) were enriched by ultracentrifugation. Hiltonol and ELANE were then loaded into LA-Exos via electroporation, and the optimal loading conditions were 400 V and 150 μF in 300 µL of buffer, as determined by measuring the fluorescence intensity of FITC-labeled ELANE and CX-rhodamine-labeled Hiltonol (Fig. S1A-1D, Fig. 1D) and nanoscale flow cytometry (Fig. 1E). Finally, the constructed HELA-Exos showed a slightly larger average diameter than the parental LA-Exos (Fig. 1F and G), exhibited increased α-LA expression (Fig. 1H), and were well dispersed in phosphate-buffered saline (PBS, pH 7.4) and 10% fetal bovine serum (FBS), with long-term stability (Fig. 1I). Thus, these results suggest that HELA-Exos may have the potential to be used in in vitro and in vivo studies.

### In vitro targeted delivery and killing capacity of HELA-Exos

To evaluate the targeting specificity of HELA-Exos, CFSE-labeled MDA-MB-231 cells were mixed with human peripheral blood mononuclear cells (PBMCs) at a 5:1 ratio, and then DiI-labeled HELA-Exos were added to this cell mixture. Compared with Texs, HELA-Exos showed a marked enrichment in MDA-MB-231 cells (53.81% ± 2.95% *vs.* 8.12% ± 2.44%), while there was no difference in PBMCs (Fig. 1J) and lung cancer A549 cells (Fig. S1E). These results indicate that HELA-Exos have excellent targeting specificity for breast cancer cells.

**Fig. 2:**
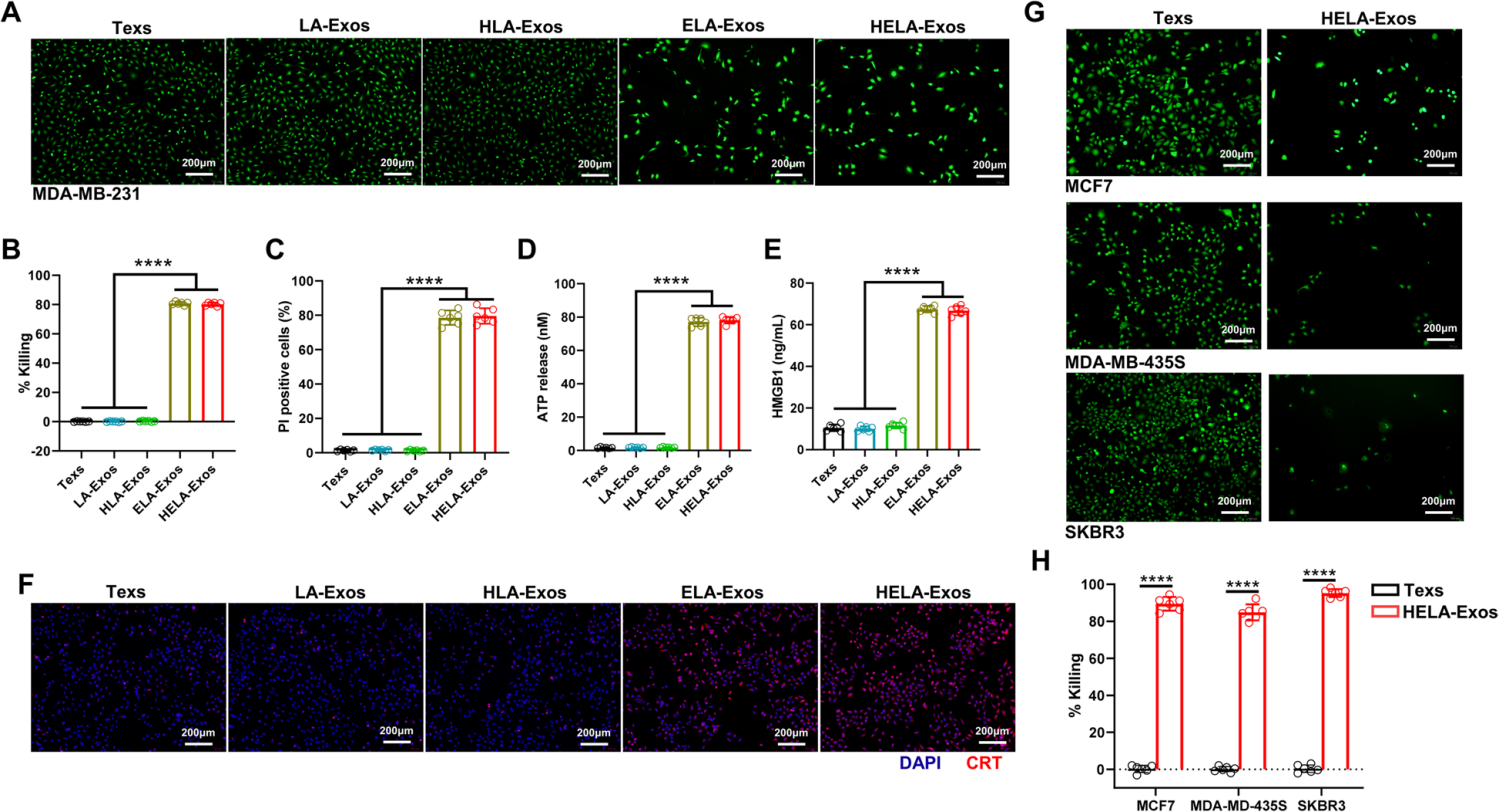
In vitro killing capacity of HELA-Exos. **A to F** MDA-MB-231 cells were treated with Texs, LA-Exos, HLA-Exos, ELA-Exos, or HELA-Exos for 48 h. **A and B** Representative images of calcein-AM fluorescence and the quantitative detection of MDA-MB-231 cell viability. Scale bar, 200 μm. The data are presented as the mean ± SD; *n* = 6. One-way ANOVA was performed for statistical analysis (****: *P* < 0.0001). **C** Apoptosis analysis by PI staining and flow cytometry. The data are presented as the mean ± SD; *n* = 6. One-way ANOVA was performed for statistical analysis (****: *P* < 0.0001). **D and E** ATP release and HMGB1 release were evaluated by ELISA. The data are presented as the mean ± SD; *n* = 6. One-way ANOVA was performed for statistical analysis (****: *P* < 0.0001). **F** The surface exposure of CRT on MDA-MB-231 cells was evaluated by CLSM. Blue: DAPI; Red: CRT. Scale bar, 200 μm. **G and H** MCF7, MDA-MB-435S, and SKBR3 cells were treated with Texs or HELA-Exos for 48 h. Representative images of calcein-AM fluorescence and the quantitative detection of cell viability in different cells. Scale bar, 200 μm. The data are presented as the mean ± SD; *n* = 6. A *t test* was performed for statistical analysis (****: *P* < 0.0001). ICD: immunogenic cell death; CRT: calreticulin; CLSM: confocal laser scanning microscopy

ELANE selectively kills a broad range of cancer cells and exhibits minimal toxicity to noncancer cells [30]. In this study, ELANE-loaded Exos (HELA-Exos and ELA-Exos) effectively killed MDA-MB-231 cells, as determined by quantifying the calcein-AM fluorescence signal (Fig. 2A and B), PI staining, and PARP1 and CASP3 expression (Fig. 2C, Fig. S2A and B). Concurrently, the levels of three hallmarks of ICD, namely, calreticulin (CRT), ATP and HMGB1, were significantly increased in HELA-Exo-treated cells compared with parental Exo-treated cells (Fig. 2D-F). These results clearly indicate that HELA-Exos induce potent ICD in breast cancer cells *in vitro*. To assess the general applicability of HELA-Exos in other subtypes of breast cancer cells, MCF7, MDA-MB-435S, and SKBR-3 cells were further treated with HELA-Exos. The results of the calcein-AM fluorescence quantitative test showed that the HELA-Exos more effectively killed MCF7, MDA-MB-435S, and SKBR3 cells compared with the parental Texs (Fig. 2G and H). These results demonstrate that HELA-Exos exhibit general killing capacity in different types of breast cancer cells.

### HELA-Exos stimulate the antigen cross-presentation activity of cDC1s and induce potent CD8^+^ T cell responses in vitro

**Fig. 3:**
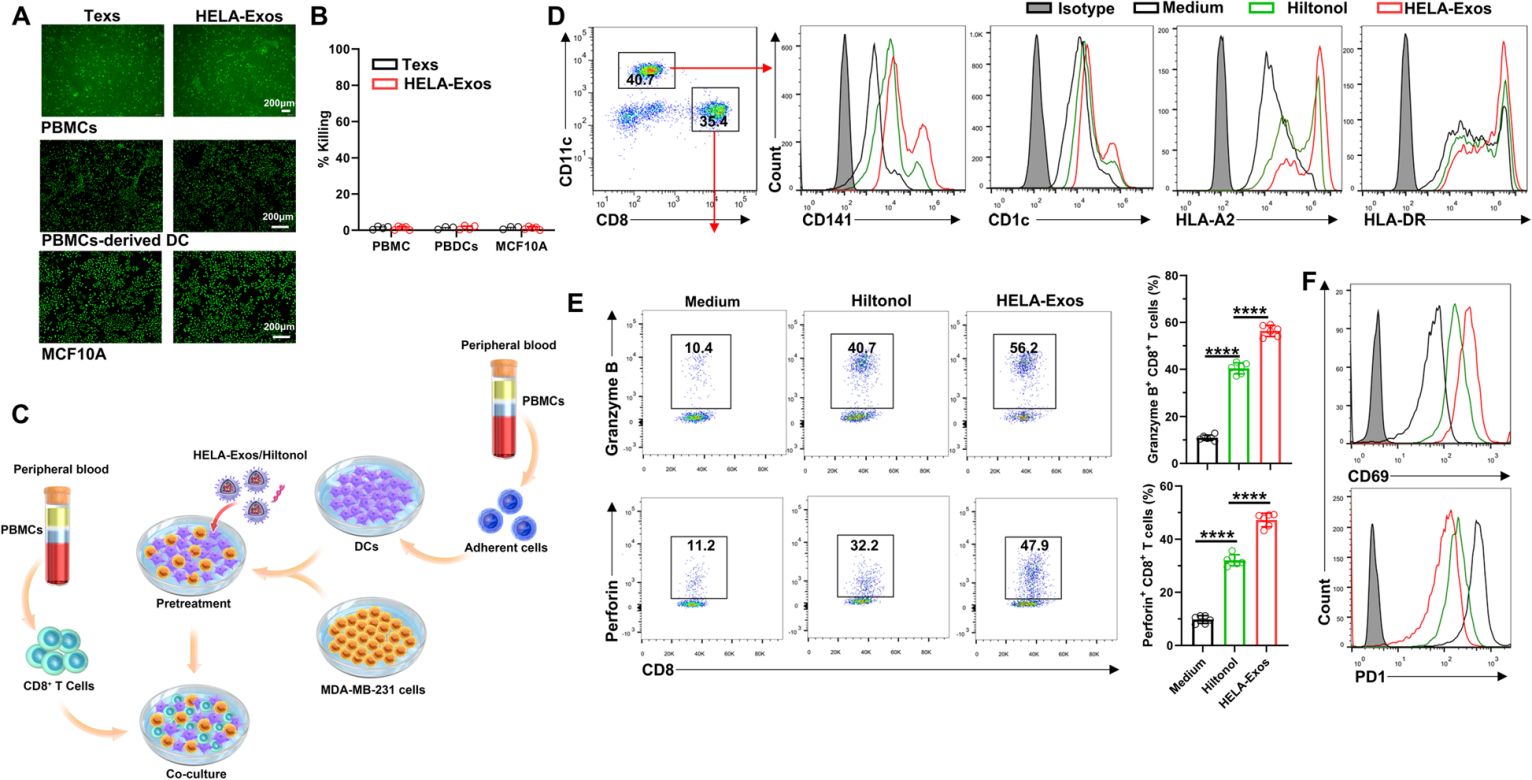
HELA-Exos stimulate the antigen cross-presentation activity of cDC1s and induce potent CD8^+^ T cell responses in vitro. **A and B** PBMCs, PBMC-derived DCs, and MCF10A cells were treated with Texs or HELA-Exos for 48 h. Representative images of calcein-AM fluorescence and the quantitative detection of cell viability in different cells. Scale bar, 200 μm. The data are presented as the mean ± SD; *n* = 6. A *t test* was performed for statistical analysis (ns: *P* > 0.05). **C** Schematic diagram of the establishment of an MDA-MB-231 cell-DC-CD8^+^ T cell coculture model. PBMC-derived DCs were cocultured with MDA-MB-231 cells and pretreated with medium, Hiltonol or HELA-Exos for 24 h. CD8^+^ T cells were added and cocultured for 48 h. **D** The cells from the coculture model were collected for flow cytometry analysis. The gating strategy for the analysis of CD11c^+^ DCs and CD8^+^ T cells was established, and the expression of CD141, CD1c, HLA-A2, and HLA-DR on DCs were measured. **E and F** The expression levels of granzyme B, perforin, CD69, and PD1 on CD8^+^ T cells were measured. The data are presented as the mean ± SD; *n* = 6. A *t test* was performed for statistical analysis (****: *P* < 0.0001)

Next, we further verified whether HELA-Exos kill normal cells before evaluating their immune activation ability. Human normal cells (PBMCs, PBMC-derived DCs, and MCF10A cells) were treated with HELA-Exos, and the results confirmed that HELA-Exos were not toxic to all human normal cells tested (Fig. 3A and B). Then, the DC-priming potential of HELA-Exos was evaluated in a cancer cell-DC-CD8^+^ T cell coculture model (Fig. 3C). Flow cytometry analysis revealed that HELA-Exo treatment induced significantly higher fluorescence intensity of CD141 (a marker of cDC1s) and HLA-A2 in DCs than Hiltonol, while CD1c (a marker of cDC2s) and HLA-DR showed no obvious difference (Fig. 3D). These results indicate that HELA-Exos are more potent in inducing maturation of the cDC1 subset than Hiltonol. cDC1s are efficient in the processing and cross-presentation of TAAs on MHC-I molecules to activate CD8^+^ T cells in breast cancer [35]. Indeed, compared to Hiltonol treatment, HELA-Exo treatment had a more robust priming effect on CD8^+^ T cells, as evidenced by higherproduction of granzyme B and perforin, increased expression of CD69 (activation marker) and lower expression of PD1 in CD8^+^ T cells (Fig. 3E and F), which induce specific cytotoxic activity against breast cells, rather than lung cancer cells or normal breast cells in vitro (Fig. S2C). Overall, these results demonstrate that the HELA-Exos selectively induce ICD in breast cancer cells and thus contribute to cDC1 maturation and CD8^+^ T cell activation *in vitro*.

### Antitumor efficacy of HELA-Exos in mice with orthotopic TNBC in vivo

We next investigated the biodistribution and tumor accumulation of HELA-Exos in vivo. CX-Rhodamine-labeled Hiltonol and HELA-Exos-DiI were administered intravenously to orthotopic tumor-bearing mice. Compared with the free CX-Rhodamine-labeled Hiltonol, a stronger fluorescence signal was detected in the tumor region of HELA-Exos-DiI-treated mice at 2 h post-injection, and it persisted to 24 h post-injection (Fig. S3A), implying an excellent targeting ability of HELA-Exos. The in vivo pharmacokinetic analysis revealed that the half-life of HELA-Exos showed no obvious changes between the first and last treatment, and the HELA-Exos exhibited superior retention in the blood compared to that of Hiltonol (8.41 ± 0.37 h *vs*. 3.71 ± 0.90 h, Fig. S3B), which demonstrates the stability of HELA-Exos in vivo*.*

**Fig. 4:**
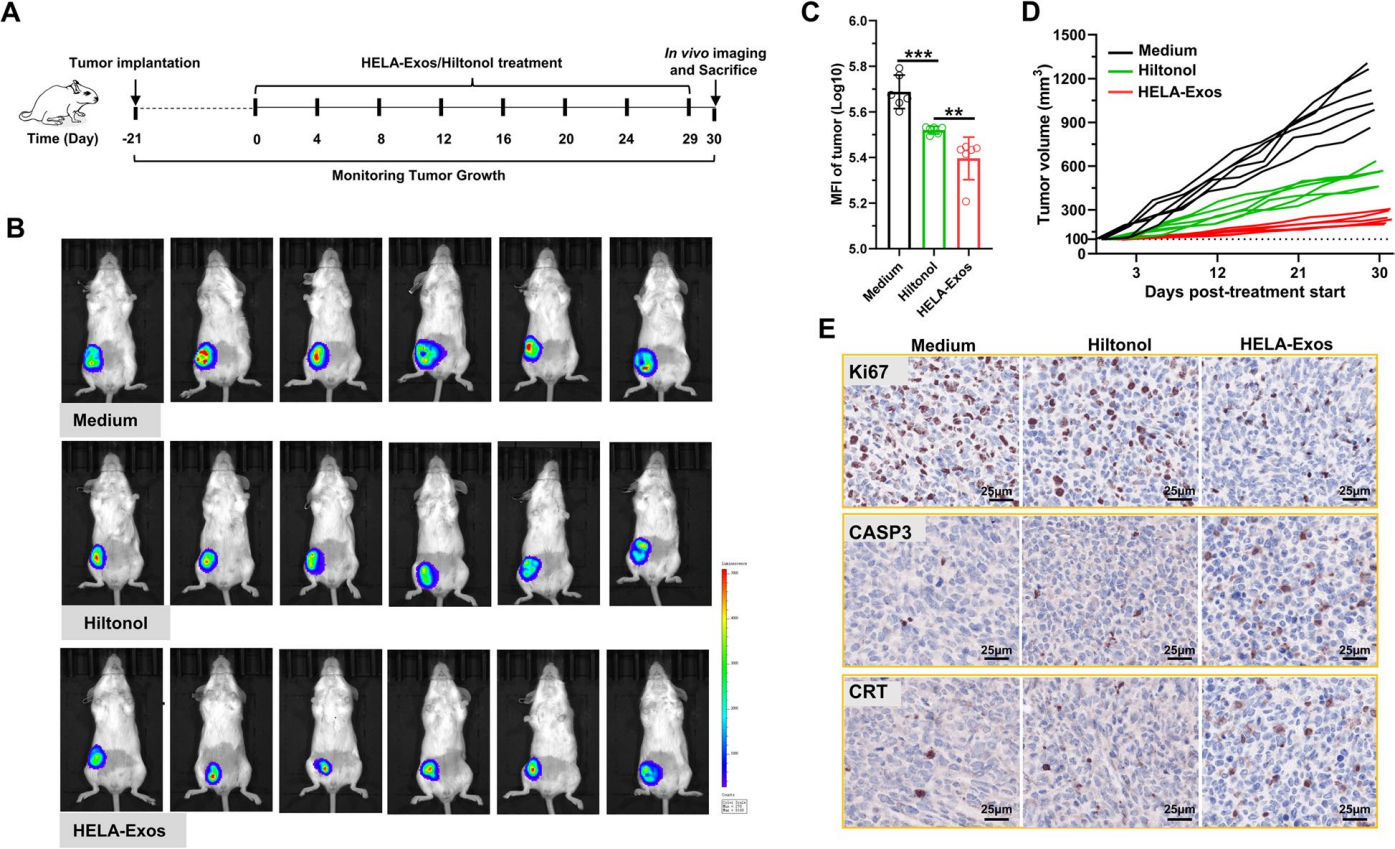
Antitumor efficacy of HELA-Exos in an orthotopic TNBC model in vivo. Establishment of a xenograft human tumor model in immunocompetent mice, as described in the methods section, and initiation of therapy. **A** Schematic diagram of the vaccine dosing regimen in Balb/c mice with orthotopic breast cancer. **B and C** In vivo tumor imaging in mice was performed using an IVIS Spectrum In Vivo Imaging System at Day 30 after initiation of therapy, and the bioluminescence intensity of the tumor was proportional to the size. The data are presented as the mean ± SD; *n* = 6. A *t test* was performed for statistical analysis (***: *P* < 0.001; **: *P* < 0.01). **D** Tumor size was measured every other day after treatment initiation. Tumor growth curves for each mouse are shown. **E** Immunohistochemical staining examination of Ki67, CASP3, and CRT in tumor tissue. Scale bar, 25 μm. MFI: mean fluorescence intensity

Encouraged by biodistribution results, the tumor inhibition of HELA-Exos was next evaluated. The therapeutic potential of the HELA-Exos was investigated after their systemic administration in an orthotopic TNBC mouse model according to the treatment regimen presented in Fig. 4A. MDA-MB-231/luc cells were inoculated into orthotopic mammary fat and allowed to grow for approximately 21 days to ~ 100 mm^3^ before administration of Hiltonol or HELA-Exos. Tumor growth was recorded by measuring the bioluminescence signal from luciferase-tagged MDA-MB-231 cancer cells (Fig. 4B). The intensity of the bioluminescence signal and tumor volume were found drastically decrease over the duration of treatment in the groups treated with Hiltonol or HELA-Exos compared with the medium control group, and more importantly, the group that received HELA-Exos had the lowest bioluminescent signal intensity and tumor volume (Fig. 4C and D). The therapeutic efficacy of the HELA-Exos was further evidenced by the fact that compared to breast tissues in the medium control and Hiltonol-treated groups, isolated breast cancer tissues exhibited the lowest Ki67 expression and the highest CASP3 and CRT expression (Fig. 4E). Thus, it was evident that HELA-Exos exhibited higher therapeutic efficacy for breast cancer than free Hiltonol. Histological examination of the major organs and biochemical analysis revealed that systemic administration of HELA-Exos did not cause severe side effects in mice (Fig. S3C and S3D). Overall, these results demonstrated that HELA-Exos possess promising therapeutic potential for breast cancer.

### HELA-Exos induce intratumoral accumulation of cDC1s and CD8^+^ T cells

**Fig. 5:**
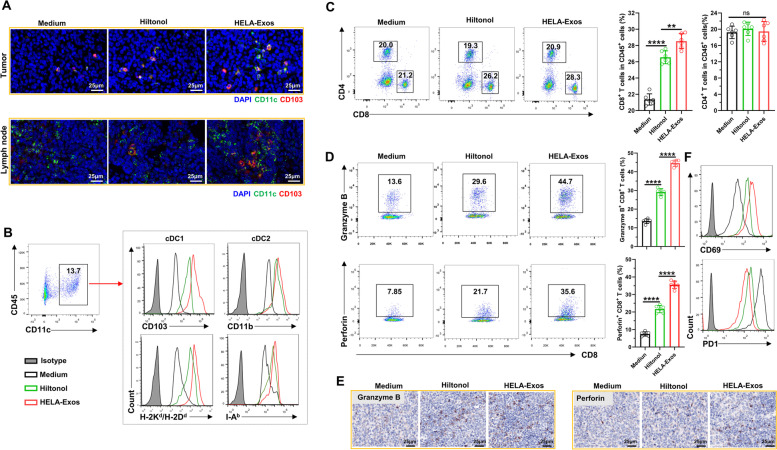
HELA-Exos induce intratumoral accumulation of cDC1s and CD8 + T cells. Balb/c mice with orthotopic breast cancer were sacrificed at day 30 after treatment initiation. Tumor tissues and draining lymph node immune infiltrates were analyzed by IF, flow cytometry and IHC. **A** Tumor tissue- and draining lymph node-infiltrating cDC1s were detected by IF. Blue: DAPI; Green: CD11c; Red: CD103. Scale bar, 25 μm. **B** Gating strategy for the analysis of CD11c^+^ DCs; the expression of CD103, CD11b, H-2K^d^/H-2D^d^ (MHC-I), and I-A^b^ (MHC-II) on DCs was measured using flow cytometry. **C** The percentages of tumor-infiltrating CD4^+^ or CD8^+^ T cells were measured using flow cytometry. **D to F** Tumor-infiltrating CD8^+^ T cells were analyzed for the expression of the cytotoxicity markers granzyme B and perforin, the activation marker CD69 and the immunosuppressive marker PD1. The data are presented as the mean ± SD; *n* = 6. *t test* and one-way ANOVA were performed for statistical analysis (****: *P* < 0.0001; **: *P* < 0.01; ns: *P* > 0.05). Scale bar, 25 μm. IF: immunofluorescence; IHC: immunohistochemistry

**Fig. 6:**
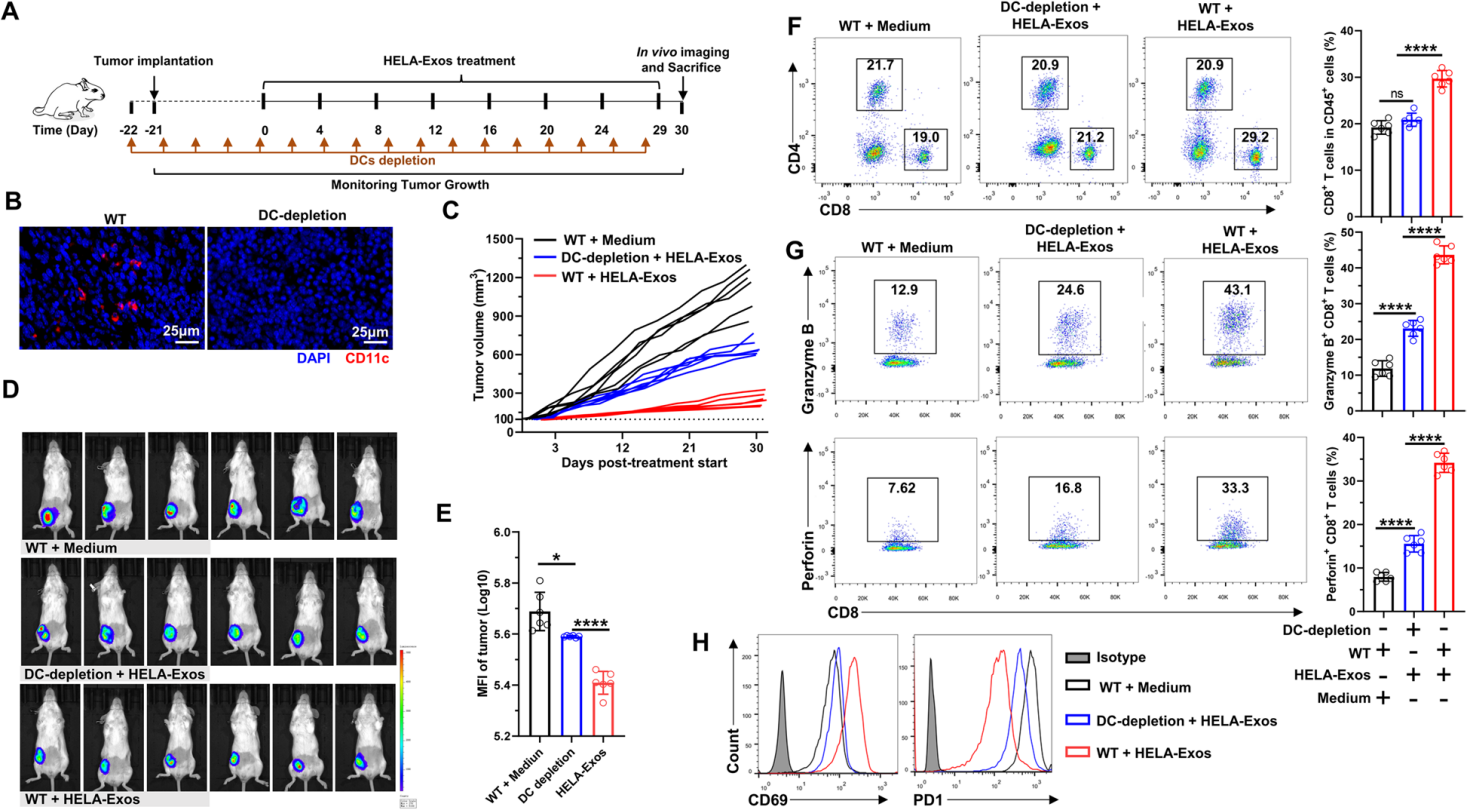
DC depletion limited the antitumor efficacy of HELA-Exos in vivo. DC depletion models were established as described in the methods section, and therapy was initiated. The mice were sacrificed at Day 30 after treatment initiation. Tumor tissue immune infiltrates were analyzed by flow cytometry. **A** Schematic diagram of the vaccine dosing regimen for DC-depleted or nondepleted BALB/c mice with orthotopic breast cancer. **B** DC depletion efficiency was confirmed by IF. Blue: DAPI; Red: CD11c. Scale bar, 25 μm. **C** Tumor size was measured every other day after treatment initiation. Tumor growth curves for each mouse are shown. **D and E** In vivo tumor imaging in mice was performed using an IVIS Spectrum In Vivo Imaging System at Day 30 after initiation of therapy, and the bioluminescence intensity of the tumor was proportional to the size. **F** The percentages of tumor-infiltrating CD4^+^ or CD8^+^ T cells were measured using flow cytometry. **G and H** Tumor-infiltrating CD8^+^ T cells were analyzed for the expression of granzyme B, perforin, CD69, and PD1 by flow cytometry. The data are presented as the mean ± SD; *n* = 6. A *t test* was performed for statistical analysis (****: *P* < 0.0001; *: *P* < 0.05 ns: *P* > 0.05)

Immune cells in isolated breast cancer tissues were further analyzed to evaluate the immune activation ability of HELA-Exos in vivo. Immunofluorescence results demonstrated that HELA-Exo treatment markedly increased the infiltration of DCs, especially the cDC1 subset of mice (CD11c^+^CD103^+^), in both the TME and draining lymph nodes, while the infiltration of macrophages was basically unchanged compared to that observed during Hiltonol treatment (Fig. S4, Fig. 5A). Flow cytometry analysis also confirmed the potent accumulation and maturation of the cDC1 subset in the TME after HELA-Exo treatment (Fig. 5B), suggesting that the targeted codelivery of an ICD inducer with a TLR3 agonist efficiently primed DCs in situ. In addition, compared to the medium control or Hiltonol treatment, HELA-Exo treatment markedly increased infiltration (Fig. 5C), granzyme B and perforin production (Fig. 5D and E), and CD69 expression and decreased PD1 expression (Fig. 5F) in specific cytotoxic CD8^+^ T cells within the TME (Fig. S5). In addition, despite demonstrating immune activation ability, repeated injections of HELA-Exos induced greatly reduced systemic inflammation relative to that induced by free Hiltonol (Fig. S6). Notably, depletion of DCs (Fig. 6A and B, Fig. S7A) significantly blocked the antitumor effects of HELA-Exos in breast cancer (Fig. 6C-E), as well as its active effects on CD8^+^ T cells (Fig. 6F-H, Fig. S7B) within the TME. Taken together, these results demonstrate that HELA-Exos can effectively promote the infiltration and maturation of the cDC1 subset in situ and boost antitumor CD8^+^ T cell immunity within the TME.

### HELA-Exos profoundly enhance cDC1 antigen cross-presentation and tumor-reactive CD8^+^ T cell generation in a patient-derived tumor organoid coculture system

**Fig. 7:**
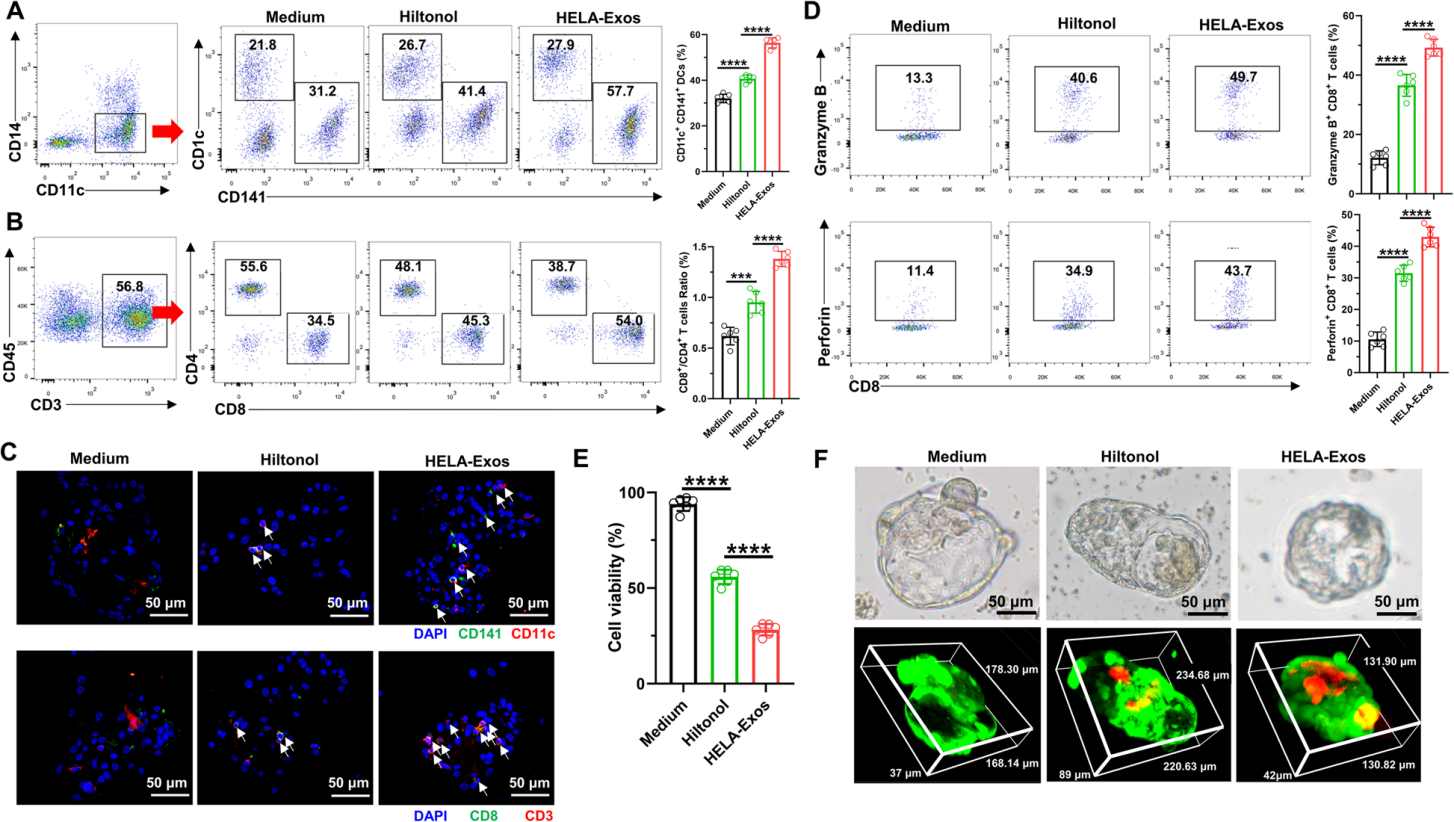
HELA-Exos profoundly enhance cDC1 antigen cross-presentation and tumor-reactive CD8 ^+^ T cell generation in a patient-derived tumor organoid coculture system. PBMCs were cocultured with autologous tumor organoids derived from breast cancer for 2 weeks and administered medium, Hiltonol or HELA-Exos during this two-week period. After two weeks of coculture, immune infiltration in organoids was evaluated by flow cytometry and IF, and the growth-inhibitory effects of HELA-Exos in organoids were evaluated by a cell viability assay and live/dead cell staining. **A** Gating strategy for analysis of CD11c^+^ DCs and the percentage of CD141^+^ cDC1s in organoids. **B** Gating strategy for analysis of CD3^+^ T cells and the percentages of CD4^+^ and CD8^+^ T cells in organoids. **C** Organoid-infiltrating CD11c^+^ CD141^+^ cDC1s and CD3^+^ CD8^+^ T cells were detected by IF. Blue: DAPI; Green: CD141 or CD8; Red: CD11c or CD3. Scale bar, 50 μm. **D** Organoid-infiltrating CD8^+^ T cells were analyzed for the expression of the cytotoxicity markers granzyme B and perforin. **E** Cell viability of organoids was measured with MTS reagent. **F** Representative bright field images of organoids from various treatment groups. The figure below shows representative three-dimensional images of organoids from various treatment groups stained with LIVE/DEAD reagents. Scale bar, 50 μm. The data are presented as the mean ± SD; *n* = 6. A *t test* was performed for statistical analysis (****: *P* < 0.0001; ***: *P* < 0.001)

To confirm the therapeutic potential of this strategy for human cancer therapy, we then tested whether HELA-Exos stimulate human T cells in patient-derived tumor organoids (PDOs). According to a previously reported protocol [36], PBMCs from breast cancer patients were cocultured with autologous PDOs for 2 weeks. In addition, Hiltonol or HELA-Exos were administered to the organoid-PBMC coculture systems (Fig. S8). Flow cytometry and immunofluorescence analysis revealed that the infiltration of CD141^+^ cDC1s and CD8^+^ T cells (Fig. 7A-C), as well as the production of granzyme B and perforin in CD8^+^ T cells (Fig. 7D), were significantly increased in HELA-Exo-treated PDOs compared to the Hiltonol- or medium-treated groups. These results suggest that HELA-Exos are effective in enhancing DC activation and the subsequent potent priming of tumor-reactive CD8^+^ T cells in autologous PDOs.

The growth-inhibitory effects of HELA-Exos in autologous PDOs were further evaluated by MTS assays and live-dead staining. Consistent with the aforementioned in vitro and in vivo observations, HELA-Exo treatment significantly inhibited organoid growth (Fig. 7E, Table S1) and induced potent cell death (Fig. 7F) in autologous PDOs. More importantly, HELA-Exos showed more potency than Hiltonol in autologous PDOs. Overall, these results suggest that HELA-Exos exhibit potent antitumor activity in breast cancer because they promote the activation of DCs in situ and subsequent tumor-reactive CD8^+^ T cell priming.

## Discussion

TNBC, a specific subtype of breast cancer characterized by lack expression of estrogen receptor (ER), progesterone receptor (PR), and human epidermal growth factor receptor 2 (HER2), makes up approximately 15% of all breast cancers [37]. Due to its special molecular phenotype, TNBC is not sensitive to endocrine therapy or molecular targeted therapy, and the efficacy of conventional postoperative adjuvant chemoradiotherapy is poor. The median survival time of TNBC after metastasis is only 13.3 months, and the recurrence rate after surgery is as high as 25% [38]. Therefore, the development of new TNBC treatment strategies has become an urgent clinical need. In the present study, we developed an in situ DC vaccine (HELA-Exos) by using engineered breast cancer-derived exosomes to codeliver the ICD inducers ELANE and Hiltonol for the treatment of breast cancer. HELA-Exos exhibit potent antitumor activity in both a mouse model and human organoids of breast cancer by promoting the activation of cDC1s in situ and thus improving the subsequent tumor-reactive CD8^+^ T cell responses.

To date, several vaccines targeting DCs are currently being tested for breast cancer in clinical trials. The DC-targeted vaccine oxidized mannan-MUC-1 dramatically decreased the recurrence rate in 31 patients with breast cancer (12.5% *vs.* 60% in patients who received placebo) at 15 years of follow-up [39]. Although many of these vaccines are promising and have been shown to be successful in breast cancer, a large population of patients still do not benefit from these therapies [40]. The potential reasons may include two factors: 1) cDC1s usually exhibit poor maturation in the TME and are thus less effective in presenting tumor antigens [16], and 2) the intrinsic tumor antigen heterogeneity in the breast cancer TME may result in limited selective pressure from tumor-infiltrating cells [41]. Compared with free Hiltonol, the HELA-Exos investigated in this study, consisting of the TLR3 agonist Hiltonol, the ICD inducer ELANE and the immunodominant antigen α-LA, effectively activated cDCs and improved antitumor CD8^+^ T cell responses. Based on these findings, HELA-Exos can potentially serve as a robust therapeutic regimen for breast cancer.

TLR3 stimulation is a potent strategy to induce the activation and maturation of cDC1s in the breast cancer TME [42, 43]. cDC1s are critical for TME immunosurveillance and preferentially infiltrate TNBCs compared to the other two subtypes, and they predict good clinical outcomes in breast cancer [35, 42]. The FDA has granted the orphan drug designation to the TLR3 agonist poly(I:C)-expressing rabies virus-based vaccine YS-ON-001 for the treatment of hepatocellular carcinoma and pancreatic cancer [44]. Thus, TLR3 agonists may represent a suitable adjuvant for the design of DC vaccines against breast cancer.

Delivery of ICD inducers, such as SR-4835 (a CDK12/13-specific inhibitor), RIG-I agonists and chemotherapeutics, as well as photothermal agents, with synthetic nanoparticles, has been confirmed to have therapeutic potential in several preclinical models of breast cancer [45–47]. Although Texs play a major role in tumor immune evasion and growth, they can be manipulated and have tumor-homing properties, as well as low in vivo toxicity and immunogenicity [48]. Therefore, we loaded Hiltonol and ELANE into breast cancer cell-derived exosomes distinct from previous synthetic nanocarriers. Moreover, ELANE is a serine protease released by human neutrophils that selectively kills cancer cells and has no toxicity to normal cells [30]. Although much work is necessary for systematic assessment of potential short-term and long-term toxicity, our small-scale pilot toxicity study, together with the safety data from clinical trials of autologous/allogenic exosomes, can alleviate safety concerns about our HELA-Exos.

Although exosome-based therapeutics hold great potential for treating multiple human diseases, the application of exosomes in drug delivery has been limited because producing sufficient quantities for in vivo use is technically challenging [49]. Currently, electroporation is the most widely used technique for exosome loading with exogenous cargo [50]. However, this approach is poorly suited for loading macromolecular proteins and inserting large nucleic acids. In the future, newly constructed approaches that overcome these shortcomings, such as “exosomes for protein loading via optically reversible protein–protein interactions” (EXPLORs) [51] and cellular nanoporation [49], should be introduced for loading exogenous cargoes into exosomes.

Autologous DC vaccines are the most investigated approaches and have been extensively investigated in more than 200 completed clinical trials but have not yet provided robust clinical benefits in large patient populations [10, 52–54]. The potential reason may be that this approach involves the ex vivo differentiation, antigen loading and maturation of autologous DCs in a controlled environment and reinfusion into patients. Thus, it is often difficult to standardize and scale up the precise conditions, such as the DC source (monocytes, CD34^+^ precursors or other subsets), the composition of the maturation cocktail, the nature of the tumor antigen (TAA or tissue-specific antigens), and the route and dose of DC vaccination [55]. Moreover, the cost is higher than that of other approaches due to the need for personalized cell therapy products. Compared to the preparation of autologous DC vaccines, it is conceivable that targeted delivery of ICD inducers and adjuvants into tumor cells to activate DCs in situ can be generalized to many other types of cancers.

In summary, we developed and optimized a formulation of tumor cell-specific exosomes (HELA-Exos) to specifically transfer the TLR3 agonist Hiltonol and the ICD inducer ELANE into tumor cells. This strategy enhances the immunogenicity of TNBC cells and indirectly activates tumor-infiltrating cDC1s in situ in both a mouse xenograft model and patient-derived tumor organoids. Beyond breast cancer, tumor-derived exosome-based delivery of ICD inducers and controllable adjuvants to tumor cells can be extended to various types of cancers.

## Methods

### Culture of cell lines

MDA-MB-231/luc cells (luciferase-expressing cells) were purchased from Guan&Dao Biological Technology Ltd. MDA-MB 231, MDA-MB-231/luc, and MDA-MB-435S cells were grown in Leibovitz's L-15 (HyClone) supplemented with 10% FBS at 37 °C in a suitable incubator without CO_2_. MCF10A cells were grown in MEGM™ BulletKit™ (Lonza) supplemented with 100 ng/mL cholera toxin. BGC823 cells, T24 cells, A549 cells, MCF7 cells, and SKBR3 cells were grown in DMEM (HyClone), THP1 cells were grown in RPMI 1640 (HyClone), the media were supplemented with 10% FBS. The cells were grown at 37 °C in the presence of 5% CO_2_. MDA-MB 231, MDA-MB-435S, MCF10A, A549, MCF7, and SKBR3 cells were kindly provided by Professor Shi Liu (Wuhan University). BGC823, T24, and THP1 cells were kindly provided by Professor Shaoping Liu (Zhongnan Hospital of Wuhan University).

### Reverse transcription qPCR analysis

RT–qPCR was performed to detect the mRNA levels of α-LA in cells. Total RNA was extracted from the cells using TRIzol (Invitrogen, Cat: 10,296,010) according to the manufacturer’s instructions. The RNA concentration and purity were measured with a NanoDrop One trace spectrophotometer (Thermo Fisher Scientific). Reverse transcription of RNA was performed with ReverTra Ace qPCR RT Master Mix (TOYOBO, Cat: FSQ-201). qPCR was performed on a StepOnePlus Real-Time PCR System (Applied Biosystems) by using a standard SYBR green assay protocol. The specificity of the qPCR products and the data were analyzed using the sequence detection software supplied with StepOne Software (Applied Biosystems). The qPCR primers used in this experiment are given in Supplementary Table 2.

### Cell transfection

The human α-LA full-length coding sequence was cloned from total RNA isolated from MDA-MB-231 cells and cloned into the lentiviral expression vector pCDH-CMV-puro (System Biosciences, USA). Human 293 T cells were seeded in a 6 cm Petri dish for 24 h, followed by cotransfection with pCDH-CMV-puro-α-LA, pMD2.G and psPAX2 plasmids at a ratio of 2:1:1 with DNA transfection reagent (Neofect, USA, Cat: TF201201). Viruses were harvested and titred 48 h later and used for subsequent MDA-MB-231 cell infection. MDA-MB-231 cells were seeded in 12-well plates and infected with α-LA-expressing lentivirus. The infection was repeated 3 times every 18 h, followed by puromycin selection (2 µg/mL). Exosomes were harvested from α-LA/MDA-MB-231 cells.

### Western blotting

Various amounts of protein were prepared from cell lysates or exosomes. The concentration of protein was detected with a BCA Protein Assay Kit (Beyotime Biotechnology, Cat: P0012S). Protein samples were separated using sodium dodecyl sulfate–polyacrylamide gel electrophoresis (SDS–PAGE) and transferred onto nitrocellulose membranes (EMD Millipore). The membranes were blocked with 5% nonfat milk at room temperature for 2 h and incubated overnight with the indicated primary antibody. Secondary antibody was added and incubated for 1 h at 37 °C. Finally, the blots were detected with an enhanced chemiluminescence kit (Meilunbio, Cat: MA0186) and analyzed with VisionWorksLS Software. The antibodies used for Western blotting are presented in Supplementary Table 3.

### Exosome preparation

Cell culture medium was sequentially centrifuged at 300 g for 10 min, 2,000 g for 10 min, and 10,000 g for 30 min. The supernatant was collected and filtered with a 0.22 μm filter, followed by ultracentrifugation at 100,000 g for 1 h to pellet exosomes. Exosome pellets were washed in a large volume of PBS and recovered by centrifugation at 100,000 g for 1 h. All centrifugation steps were carried out at 4 °C. The total protein concentration of exosomes was quantified with a BCA Protein Assay Kit (Beyotime Biotechnology, Cat: P0012S).

### Preparation of HELA-Exos

ELANE and Hiltonol were loaded into exosomes to fabricate the vaccine by using an electroporation system (Bio–Rad Gene Pluser Xcell). ELANE was labeled with FITC with the EZLabel™ Protein FITC Labeling Kit (Biovision, Cat: K832-5*1EA), and Hiltonol was labeled with CX-Rhodamine with the Label IT™ Nucleic Acid Labeling Kit, CX-Rhodamine (Mirus Bio, Cat: MIR3100) according to the manufacturer’s instructions. FITC-labeled ELANE or CX-rhodamine-labeled Hiltonol was dispersed in pure water at various concentrations, and the fluorescence intensity was measured at 519 nm or 597 nm to generate a fluorescence standard curve for the empirical optimization of the electroporation protocol. A series of electroporation parameters were set, including capacitance, voltage and buffer volume. Exosomes, ELANE, and Hiltonol were mixed in electroporation buffer and electroporated in a 4 mm cuvette. After electroporation, the exosomes were spun down at 100,000 g for 1 h. Then, they were lysed in pure water, and fluorescence was assayed at 519 nm and 597 nm on a fluorescent plate reader for quantification of ELANE and Hiltonol based on the fluorescence standard curve, ultimately yielding the optimal electroporation parameters. The optimal loading efficiencies of ELANE and Hiltonol were 12.45% and 21.31%, respectively.

Finally, vaccines (HLA-Exos, ELA-Exos, and HELA-Exos) were fabricated with electroporation parameters of 400 V and 150 μF in 300 µL of buffer. After electroporation, exosomes were washed two times in PBS with 100,000 g, centrifuged for 1 h and resuspended in medium for follow-up research. The loaded ELANE or Hiltonol was quantified according to the loading efficiency.

### Nanoscale flow cytometry of exosomes

The exosomes loaded with FITC-labeled ELANE and CX-Rhodamine-labeled Hiltonol were analyzed using a A50-Micro Plus Nanoscale Flow Cytometer (Apogee FlowSystems Inc.) equipped with 70 mW 405 nm (violet), 53 mW 488 nm (blue) and 73 mW 639 nm (red) lasers. The parameters in the control panel were set to a sheath pressure of 150 mbar and a number of flush cycles of 3. A sample flow rate of 1.5 µL/min was used for all measurements, and the time of acquisition was held constant for all samples at 60 s to yield enough events. An illumination wavelength of 405 nm (70 mW) was used to detect scattered light. Before sample analysis, calibration of the flow cytometer was performed using silica nanoparticles (Apogee FlowSystems Inc.). Silica nanoparticles and fluorescent polystyrene nanoparticles were used to evaluate the sensitivity and resolution of flow cytometry scattered light and fluorescence. The data were analyzed using FlowJo Software.

### Characterization of the morphology and diameter of HELA-Exos

The morphology of all the nanoparticles was characterized using transmission electron microscopy (HT7700, Hitachi). Negative staining TEM images were recorded after staining the samples with uranyl acetate (3%). The diameters of all the nanoparticles were analyzed by dynamic light scattering (DLS) with a particle sizer (Zetasizer Nano ZS, Malvern). For the stability analysis, HELA-Exos were dispersed in PBS (pH 7.4) and 10% FBS for one week, and the diameter was recorded every day.

### Targeting of HELA-Exos

Exosomes were labeled with DiI (Beyotime Biotechnology, Cat: C1991S) according to the manufacturer’s instructions. Briefly, purified exosomes from MDA-MB-231 or α-LA/MDA-MB-231 cells were incubated in the presence of 5 mM DiI for 15 min at 37 °C and then ultracentrifuged at 100,000 g for 1 h to remove the unbound dye. After being washed twice in PBS with centrifugation at 100,000 g for 1 h, Texs-DiI and LA-Exos-DiI were collected, and LA-Exos-DiI was electroporated to prepare HELA-Exos-DiI.

MDA-MB-231 cells were collected and labeled with CFSE (Beyotime Biotechnology, Cat: C0051) according to the manufacturer’s instructions. The PBMC fraction was isolated from peripheral blood by Ficoll-Paque density gradient separation and mixed with MDA-MB-231 cells at a ratio of 5:1 to establish a coculture model. The coculture model was incubated with Texs-DiI or HELA-Exos-DiI for 2 h at 37 °C and then stained with a Pacific Blue anti-human CD45 antibody (Biolegend, Cat: 304,022) for 30 min at 4 °C to mark PBMCs. The targeting capacity of HELA-Exos to MDA-MB-231 cells was detected by flow cytometry (BD FACSAria III). For the CLSM assay, MDA-MB-231 cells or A549 cells were seeded in 12-well plates for 24 h and then coincubated with Texs-DiI or HELA-Exos-DiI for 2 h at 37 °C. After rinsing with PBS twice and staining with DAPI (Sigma–Aldrich, Cat: D9542) for 15 min, the fluorescence emission of DAPI and DiI was measured to determine the uptake efficiency of MDA-MB-231 cells or A549 cells.

### Preparation of human PBMC-derived DCs

To prepare human PBMC-derived DCs, human monocytes were enriched by plastic adherence of PBMCs in a 100 mm Petri dish at 37 °C and 5% CO_2_. After 2 h of incubation, the nonadherent cells were reserved to sort CD8^+^ T cells with a Naive CD8^+^ T Cell Isolation Kit (Miltenyi Biotec, Cat: 130–093-244) for subsequent experiments. The remaining adherent cells were cultured in medium containing 100 ng/mL human GM-CSF (PeproTech, Cat: AF-300–03-50UG) and 20 ng/mL human IL-4 (InvivoGen, Cat: rcyec-hil4). After 48 h, the medium was refreshed, and 10 ng/mL TNF-α (InvivoGen, Cat: rcyc-htnfa) was added. On day 3, the human DC suspension was harvested.

### Viability and ICD evaluation of cells treated with HELA-Exos in vitro

Cancer cells (MDA-MB-231, MCF7, MDA-MB-435S, SKBR3) or noncancer cells (PBMCs, PBMC-derived DCs, MCF10A) were plated in complete growth media. Cells were washed with serum-free DMEM and treated with various vaccines (Texs, LA-Exos, HLA-Exos, ELA-Exos, and HELA-Exos; each vaccine dose contained or did not contain 3 μg ELANE or 20 μg Hiltonol) for 48 h, and cell viability was assessed using several methods:

1. Calcein-AM assay
  Forty-eight hours after treatment, cells were incubated with calcein-AM (Invitrogen, 4 ng/mL, Cat: C1430) and washed with serum-free DMEM. For representative images, the fluorescence emission of calcein-AM was collected by CLSM. For the quantitative detection of cell viability, fluorescence was measured at 495 nm/516 nm using a SpectraMax Microplate Reader (Molecular Devices).
2. Cleaved-CASP3 and cleaved-PARP1 analyses
  Forty-eight hours after treatment, the cells were washed with PBS and lysed, and the concentration of protein was detected with a BCA Protein Assay Kit (Beyotime Biotechnology, Cat: P0012S). The cleaved CASP3/CASP3 and cleaved PARP1/PARP1 levels in treated MDA-MB-231 cells were measured by Western blotting.
3. ATP and HMGB1 detection
  Forty-eight hours after treatment, the cell culture supernatant of MDA-MB-231 cells was collected and evaluated using an ATP Assay Kit (Sigma–Aldrich, Cat: 213-579-1) and a Human HMGB1 ELISA Kit (Genie, Cat: HUFI00660) according to the manufacturer’s instructions.
4. Cell immunofluorescence staining to detect CRT exposure
  Forty-eight hours after treatment, the cells were washed with PBS and fixed with 4% paraformaldehyde for 15 min at room temperature. Next, the cells were permeabilized with 0.1% Triton X-100 PBS for 10 min and cultured with blocking buffer containing 5% bovine serum albumin (BSA) for 1 hour at room temperature. Then, the cell samples were incubated overnight with an anti-calreticulin antibody at 4 °C, washed three times with PBS, and incubated in secondary antibody for 1 hour at room temperature. Finally, the cells were stained with DAPI, washed with PBS, and imaged using a confocal laser scanning microscope (Leica).

### Evaluation of the immune activation ability of HELA-Exos in vitro

MDA-MB-231 cells were collected by trypsin digestion and coculture with PBMC-derived DCs that were harvested as described previously at a ratio of 1:1. The cells were washed with serum-free DMEM and pretreated with medium, Hiltonol (20 μg), or HELA-Exos (containing 3 μg ELANE and 20 μg Hiltonol) for 24 h. Naive CD8^+^ T cells that were sorted as described above were cultured in medium containing 150 U/ml recombinant human IL-2. Twenty-four hours after pretreatment, naive CD8^+^ T cells were added to the coculture system, and coculture was continued for 48 h. Forty-eight hours later, the cells were collected for flow cytometry analysis. To detect the specific killing efficacy of CD8^+^ T cells, CD8^+^ T cells were harvested and incubated with target cells (MDA-MB-231, MCF7, A549, and MCF10A cells) at a ratio of 10:1 in 96-microwell plates at 37 °C and 5% CO_2_ for 72 h as the reactive cell group. Concurrently, the pure CD8^+^ T cell group, pure target cell control group and pure culture solution blank control group were established. In the last 4 h, 10 µL CCK8 solution (Beyotime Biotechnology, Cat: C0039) was added. The OD value indicating the absorbance of enzyme-labeled cells was detected with an instrument at 570 nm. The following formula was used to calculate the killing rate: killing rate = [1-(OD value of reactive cell well-OD value of effector cell well)/(OD value of target cell well)] × 100%.

### Flow cytometry

Antibodies for flow cytometry analysis were purchased from BioLegend. For the flow cytometry analysis of surface markers, the cells were stained on ice with fluorescence-conjugated antibodies for 30 min according to the manufacturer’s instructions. For the staining of intracellular markers, such as granzyme B, the cells were treated with brefeldin A (InvivoGen, Cat: inh-bfa) at 37 °C for 6 h. Cells were stained with antibodies against surface markers and then fixed and permeabilized using an intracellular staining kit (BD Biosciences, Cat: 559,302, 559,311) at 4 °C for 30 min and then stained with target intracellular antibody at 4 °C for 30 min. Stained cells were finally evaluated by flow cytometry and analyzed using FlowJo Software. The antibodies used for flow cytometry are presented in Supplementary Table 3.

### Tumor-bearing mice and treatment

BALB/c mice were raised in the animal facility of the Experimental Animal Center, Zhongnan Hospital of Wuhan University, under sterile conditions in air-filtered containers. The establishment of xenograft human tumor models in immunocompetent mice was conducted according to a previous report [56]. For depletion of CD11c^+^ DCs, mice were intraperitoneally injected with 200 μg purified anti-mouse CD11c mAb (Biolegend, Cat: 117,302) the day before the tumor challenge, and the injections were repeated every 2 to 3 days. Depletion efficiency was confirmed by immunofluorescence assays and flow cytometry.

A total of 5.0 × 10^6^ MDA-MB-231/luc cells in a volume of 50 µL were implanted into the #4 mammary fat pad on Day -21. Cells were maintained on wet ice during implantation. Prior to treatment initiation on day 0, the mice were randomly allocated to groups, and treatment began at a mean tumor burden of 100 mm^3^ (range of group means, 96–104 mm^3^). Medium, Hiltonol (60 μg/dose), or HELA-Exos (containing 12 μg ELANE and 60 μg Hiltonol/dose) were administered intravenously twice per week for 4 weeks.

### In vivo pharmacokinetics and biodistribution

A total of 5.0 × 10^6^ MDA-MB-231/luc cells in a volume of 50 µL were implanted into the mammary fat pad. On 15th day after the tumor inoculation, the mice were intravenous injected with medium, CX-Rhodamine-labeled Hiltonol (60 μg/dose), or HELA-Exos-DiI (containing 12 μg ELANE and 60 μg Hiltonol/dose). At different time points, in vivo fluorescence imaging was performed using Bruker Xtreme BI system. At different time points, 20 μL blood were harvested from the tail veins. The harvested samples were diluted with 30 μL PBS and the concentration of Hiltonol or HELA-Exos were determined by measuring the Mean Fluorescence Intensity (MFI) of CX-Rhodamine or DiI on a SpectraMax Microplate Reader (Molecular Devices). The serum of untreated mice was served as background control for subtraction.

### Monitoring of tumor growth and collection of tumor tissues

To measure tumor growth, mice were imaged using an IVIS Spectrum In Vivo Imaging System. Mice were injected with 150 mg/kg D-luciferin intraperitoneally. Mice were placed in an imaging chamber, and under 1–2% isoflurane gas anesthesia, they were imaged in the supine position. Bioluminescence was measured and superimposed on photographic images of the mice. Images were analyzed using Living Image 4.3.1. The breast signal was calculated using fixed-volume regions of interest to estimate the tumor burden, and total flux was calculated.

During the above antitumor experiment in vivo, tumor volumes were measured 2 to 3 times a week using a caliper and calculated using the equation V (mm^3^) = 1/2 × L × W^2^, where L (length) is the largest diameter and W (width) is the smallest diameter. We obtained tumor growth curves of individual mice in various treatment groups.

Tumor-bearing mice were sacrificed at day 30 (1 day after the last administration) after treatment. Serum samples were collected from mice before sacrifice, and the tumor tissues, draining lymph nodes, and major organs (heart, lung, liver, spleen, and kidney) were harvested for further research. The levels of serum IL-6 (Biovision, Cat: K4144-100), IL-12 (Genie, Cat: MOFI01240) and TNF-α were evaluated with ELISA kits (Genie, Cat: MOFI00104). Major organs were stained with HE. The levels of serum alanine transaminase (ALT), aspartate aminotransferase (AST), blood urea nitrogen (BUN), and creatinine (CREA) were evaluated with a Liver or Renal Function Activity Assay Kit (Servicebio, Cat: GM1102).

### Tumor immunohistochemistry and immunofluorescence

Tumor-bearing mice were sacrificed at day 30 (1 day after the last administration) after treatment. Tumor tissues and draining lymph nodes were fixed in 4% paraformaldehyde in PBS for 24 h, embedded in paraffin blocks, and sectioned (5 μm). Slides were stained with anti-mouse primary antibodies. Signals were developed using the Vectastain Elite ABC Universal plus kit, peroxidase (Vector Laboratories, Cat: PK-8200) or fluorescently labeled secondary antibodies. Cell nuclei were labeled with hematoxylin or DAPI. An Olympus IX81 fluorescence microscope was used to photograph representative peroxidase staining samples or fluorescence samples. The antibodies used for immunohistochemistry and immunofluorescence are presented in Supplementary Table 3.

### Tumor immune cell analysis by flow cytometry

Tumor-bearing mice were sacrificed at day 30 (1 day after the last administration) after treatment. The tumor tissue was cut into small pieces to generate a single-cell suspension with a Mouse Tumor Dissociation Kit (Miltenyi Biotec, Cat: 130–096-730) and gentleMACS™ Dissociators (Miltenyi Biotec), as instructed in the manufacturer’s protocol. If a visible red pellet was present after the last centrifugation of the digestion process, erythrocytes were lysed in 2 mL Red Blood Cell Lysis Solution (Miltenyi Biotec, Cat: 130–094-183) for 5 min at room temperature and centrifuged at 400 g. A Dead Cell Removal Kit (Miltenyi Biotec, Cat: 130–090-101) was used to eliminate dead cells from tissue preparations. Cells were labeled with various antibodies and analyzed by flow cytometry. The specific killing efficacy of CD8^+^ T cells was determined with a CCK-8 kit.

### Breast cancer organoid culture

Breast cancer organoids were established essentially as described in Norman Sachs et al. [57]. Tumor tissue derived from surgical resection was cut into small pieces to generate a single-cell suspension with a Human Tumor Dissociation Kit (Miltenyi Biotec, Cat: 130–095-929) and gentleMACS™ Dissociators (Miltenyi Biotec), as instructed in the manufacturer’s protocol. If a visible red pellet was present after the last centrifugation of the digestion process, erythrocytes were lysed in 2 mL Red Blood Cell Lysis Solution (Miltenyi Biotec, Cat: 130–094-183) for 5 min at room temperature and centrifuged at 400 g. A Dead Cell Removal Kit (Miltenyi Biotec, Cat: 130–090-101) was used to eliminate dead cells from tissue preparations. The cell pellet was resuspended in 10 mg/mL cold Cultrex Reduced Growth Factor Basement Membrane Matrix, Type 2 (BME 2) (Trevigen, Cat: 3533–001-02), and 40 μL drops of BME-cell suspension were allowed to solidify on prewarmed 24-well suspension culture plates at 37 °C for 20 min. Upon completion of gelation, 400 μL of human breast cancer organoid medium was added to each well, and the plates were transferred to humidified 37 °C, 5% CO_2_ incubators. The medium was changed every 4 days. In the first two weeks of organoid culture, primocin (InvivoGen, Cat: ant-pm-05) was added to prevent microbial contamination. The human breast cancer organoid medium recipe is provided in Supplementary Table 4.

### Organoid-PBMC coculture system

The organoid-PBMC coculture system was established according to the protocol provided by Krijn K. Dijkstra et al. [58]. The T cell medium was composed of RPMI 1640 supplemented with 2 mM Ultraglutamine I, 1% penicillin/streptomycin and 10% male human AB serum (SeraCare, Cat: 1810–0001). Tumor organoids were dissociated into single cells with TrypLE Express and resuspended in T cell medium. PBMCs were seeded at a density of 10^5^ cells/well, stimulated with single cell-dissociated organoids at a 20:1 effector:target ratio in the presence of 150 U/ml recombinant human IL-2 (PeproTech, Cat: 200–02), and cocultured for 2 weeks in T cell medium. The coculture system was treated with medium, Hiltonol (20 μg/dose), or HELA-Exos (containing 3 μg ELANE and 20 μg Hiltonol/dose). Half of the medium, including IL-2, Hiltonol or HELA-Exos, was replaced 2 to 3 times per week. Every week, the PBMCs were restimulated with fresh tumor organoids.

### Evaluation of the immune activation and antitumor efficacy of HELA-Exos in a coculture system

PBMCs were cocultured with autologous tumor organoids derived from breast cancer for 2 weeks and administered medium, Hiltonol or HELA-Exos. After two weeks of coculture, immune infiltration in the organoids was evaluated by flow cytometry and immunofluorescence, and the growth-inhibitory effects of HELA-Exos on organoids were evaluated via a cell viability assay and live/dead cell staining.

For flow cytometry, organoids were dissociated into single cells using TrypLE Express, and all the cells were collected for flow cytometry analysis. For immunofluorescence imaging, organoids were removed from the coculture system on day 14 and fixed with 4% paraformaldehyde for 30 min. After fixation, the spheroids were transferred to tissue culture dishes and embedded in 4% agarose. Pre-embedded agarose spheroids were embedded in paraffin after isopropanol and acetone dehydration. Four-micrometer-thick sections were cut and adhered to poly-L-lysine-coated glass slides. The sections were incubated with the primary antibodies in a humidified chamber at 4 °C overnight. Then, the sections were incubated for 1 h with secondary antibodies. The cells were counterstained with DAPI for 5 min. The sections were imaged to observe fluorescence at 488 nm, 594 nm, and 380 nm with an Olympus IX81 microscope. The antibodies used for immunofluorescence are presented in Supplementary Table 3.

The cell viability of organoids was evaluated with an MTS assay as instructed in the manufacturer's protocol. The MTS Cell Proliferation Colorimetric Assay Kit (Biovision, Cat: K300) was used, with quantification at 490 nm on a plate reader. A live-dead assay for the organoids was performed by exposing treated organoids to the Live-Dead Cell Staining Kit (Biovision, Cat: K501) solution as instructed in the manufacturer’s protocol. The organoids were incubated in live-dead solution for 20 min at 37 °C and then imaged immediately by CLSM.

### Statistical analysis

All data are reported as the mean values ± SDs. Statistical analyses were carried out using GraphPad Prism software version 8.0. Comparisons between two groups were performed using an unpaired two-tailed Student’s *t test*. One-way ANOVA was used for comparisons of more than two groups. *P* < 0.05 was considered to indicate statistical significance.

## Supplementary Information

**Additional file 1: Figure S1.** Electroporation protocol optimization and targeting of HELA-Exos. (A and B) FITC-labeled ELANE or CX-rhodamine-labeled Hiltonol dispersed in pure water at various concentrations was applied for calibration. The fluorescence standard curve of ELANE or Hiltonol was generated by measuring the fluorescence intensity at 519 nm and 597 nm. (C and D) FITC-labeled ELANE and CX-Rhodamine-labeled Hiltonol were electroporated with exosomes in 200 µl of buffer at the settings shown on the x-axis, and ELANE and Hiltonol loading was determined based on the fluorescence standard curve. The data are presented as the mean ± SD; n = 3. (E) The cellular uptake of MDA-MB-231 cells or A549 cells after incubation with Texs-DiI or HELA-Exos-DiI for 2 h was evaluated by CLSM. Blue: DAPI. Red: DiI. Scale bar, 25 μm. CLSM: confocal laser scanning microscopy. **Figure S2.** Apoptosis analysis and specific cytotoxic activity of CD8+ T cells. (A) Gating strategy for analysis of MDA-MB-231 cells; the percentages of PI-positive cells were measured. (B) Apoptosis analysis. Images of western blots for cleaved CASP3/CASP3 and cleaved PARP1/PARP1 in MDA-MB-231 cells. (C) CD8+ T cells were harvested from various treatment groups and incubated with target cells (MDA-MB-231, MCF7, A549, and MCF10A cells), and cytotoxicity was detected with a CCK-8 kit. The data are presented as the mean ± SD; n = 6. t test and one-way ANOVA were performed for statistical analysis (****: P < 0.0001; *: P < 0.05; ns: P > 0.05). **Figure S3.** Fluorescence imaging, pharmacokinetic curves, and safety of HELA-Exos in vivo. (A) In vivo fluorescence imaging in orthotopic MDA-MB-231 tumor-bearing mice at 2, 12, and 24 h following injection with CX-Rhodamine-labeled Hiltonol and HELA-Exos-DiI. (B) In vivo pharmacokinetic curves of Hiltonol and HELA-Exos. (C) Pathological examination of the major organs (the lung, heart, liver, spleen, and kidney). The tissue sections were stained with H&E. Scale bar, 100 μm. (D) The levels of the serum biochemical parameters ALT, AST, BUN, and CREA. The data are presented as the mean ± SD; n = 6. One-way ANOVA was performed for statistical analysis (ns: P > 0.05). **Figure S4.** APCs were recruited to the tumor microenvironment after HELA-Exo treatment. Balb/c mice with orthotopic breast cancer were sacrificed at day 30 after treatment initiation. Tumor-infiltrating DCs (CD11c+) and macrophages (F4/80+) were detected by IF. Blue: DAPI; Green: F4/80; Red: CD11c. Scale bar, 25 μm. IF: immunofluorescence. **Figure S5.** Specific cytotoxic activity of CD8+ T cells from the tumor microenvironment of Balb/c mice with orthotopic breast cancer. Balb/c mice with orthotopic breast cancer were sacrificed at day 30 after treatment initiation. The tumor tissue was cut into small pieces to generate a single-cell suspension. CD8+ T cells were sorted and incubated with target cells (MDA-MB-231, MCF7, A549, and MCF10A cells), and cytotoxicity was detected with a CCK-8 kit. The data are presented as the mean ± SD; n = 6. A t test and one-way ANOVA were performed for statistical analysis (****: P < 0.0001; **: P < 0.01; ns: P > 0.05). **Figure S6.** Reduced systemic inflammatory toxicity of HELA-Exos. Balb/c mice with orthotopic breast cancer were sacrificed at day 30 after treatment initiation. The serum levels of the cytokines IL-6, IL-12, and TNF-α. The data are presented as the mean ± SD; n = 6. A t test was performed for statistical analysis (****: P < 0.0001; ns: P > 0.05). **Figure S7.** DC depletion efficiency in Balb/c mice and specific cytotoxic activity of CD8+ T cells. (A) Gating strategy for the analysis of DCs; the percentages of CD11c+ DCs in the lymph nodes and spleen were measured. (B) DC-depleted or nondepleted Balb/c mice with orthotopic breast cancer were sacrificed at Day 30 after treatment initiation. The tumor tissue was cut into small pieces to generate a single-cell suspension. CD8+ T cells were sorted and incubated with target cells (MDA-MB-231, MCF7, A549, and MCF10A cells), and cytotoxicity was detected with a CCK-8 kit. The data are presented as the mean ± SD; n = 6. A t test and one-way ANOVA were performed for statistical analysis (****: P < 0.0001; *: P < 0.05; ns: P > 0.05). **Figure S8.** Tumor organoids were established from tumor tissue, and PBMCs were isolated from peripheral blood before the start of coculture. On the day of coculture, organoids were isolated from Geltrex, dissociated into single cells and plated together with PBMCs in the presence of vaccines. After 1 week of coculture, PBMCs were re-stimulated with tumor cells from organoids. After two weeks of coculture, immune infiltration in organoids was evaluated by flow cytometry and IF, and the growth-inhibitory effects in organoids of HELA-Exos were evaluated by a viability assay and live/dead cell staining.

**Additional file 2: Table 1.** Patient information for had established tumor organoids.

**Additional file 3: Table 2.** List of primers used.

**Additional file 4: Table 3.** List of antibodies for this study.

**Additional file 5: Table 4.** Human breast cancer organoids medium recipe.

## Data Availability

The original contributions presented in the study are included in the article/Supplementary Material, further inquiries can be directed to the corresponding authors.

## Abbreviations

DCs: Dendritic cells
HELA-Exos: Hiltonol-ELANE-α-LA-engineered exosomes
ICD: Immunogenic cell death
ELANE: Human neutrophil elastase
TLR3: Toll-like receptor 3
TNBC: Triple-negative breast cancer
TME: Tumor microenvironment
DAMPs: Damage-associated molecular patterns
cDC1s: Type 1 conventional dendritic cells
cDC2s: Type 2 conventional dendritic cells
PBMCs: Peripheral blood mononuclear cells
APCs: Antigen-presenting cells
TAAs: Tumor-associated antigens
Texs: Tumor-derived exosomes
α-LA: α-Lactalbumin
Exos: Exosomes
PBS: Phosphate-buffered saline
FBS: Fetal bovine serum
CRT: Calreticulin
PDOs: Patient-derived tumor organoids
ER: Estrogen receptor
PR: Progesterone receptor
HER2: Human epidermal growth factor receptor 2
DLS: Dynamic light scattering
CLSM: Confocal laser scanning microscopy
MFI: Mean fluorescence intensity
CASP3: Caspase 3
