# Supplementary material for: Engineered exosomes as an in situ DC-primed vaccine to boost antitumor immunity in breast cancer
Source: Mol Cancer. 2022 Feb 11;21:45. doi: 10.1186/s12943-022-01515-x (PMC8831689; doi:10.1186/s12943-022-01515-x)
Supplement: Supplementary file 1 — Additional file 1: Figure S1. Electroporation protocol optimization and targeting of HELA-Exos. (A and B) FITC-labeled ELANE or CX-rhodamine-labeled Hiltonol dispersed in pure water at various concentrations was applied for calibration. The fluorescence standard curve of ELANE or Hiltonol was generated by measuring the fluorescence intensity at 519 nm and 597 nm. (C and D) FITC-labeled ELANE and CX-Rhodamine-labeled Hiltonol were electroporated with exosomes in 200 µl of buffer at the settings shown on the x-axis, and ELANE and Hiltonol loading was determined based on the fluorescence standard curve. The data are presented as the mean ± SD; n = 3. (E) The cellular uptake of MDA-MB-231 cells or A549 cells after incubation with Texs-DiI or HELA-Exos-DiI for 2 h was evaluated by CLSM. Blue: DAPI. Red: DiI. Scale bar, 25 μm. CLSM: confocal laser scanning microscopy. Figure S2. Apoptosis analysis and specific cytotoxic activity of CD8+ T cells. (A) Gating strategy for analysis of MDA-MB-231 cells; the percentages of PI-positive cells were measured. (B) Apoptosis analysis. Images of western blots for cleaved CASP3/CASP3 and cleaved PARP1/PARP1 in MDA-MB-231 cells. (C) CD8+ T cells were harvested from various treatment groups and incubated with target cells (MDA-MB-231, MCF7, A549, and MCF10A cells), and cytotoxicity was detected with a CCK-8 kit. The data are presented as the mean ± SD; n = 6. t test and one-way ANOVA were performed for statistical analysis (****: P < 0.0001; *: P < 0.05; ns: P > 0.05). Figure S3. Fluorescence imaging, pharmacokinetic curves, and safety of HELA-Exos in vivo. (A) In vivo fluorescence imaging in orthotopic MDA-MB-231 tumor-bearing mice at 2, 12, and 24 h following injection with CX-Rhodamine-labeled Hiltonol and HELA-Exos-DiI. (B) In vivo pharmacokinetic curves of Hiltonol and HELA-Exos. (C) Pathological examination of the major organs (the lung, heart, liver, spleen, and kidney). The tissue sections were stained with H&E. Scale bar, 100 μm [file 12943_2022_1515_MOESM1_ESM.zip › Revised Supplementary figure legends.docx]

**Supplementary figure legends**

**Figure S1. Electroporation protocol optimization and targeting of HELA-Exos.**

**(A and B)** FITC-labeled ELANE or CX-rhodamine-labeled Hiltonol dispersed in pure water at various concentrations was applied for calibration. The fluorescence standard curve of ELANE or Hiltonol was generated by measuring the fluorescence intensity at 519 nm and 597 nm. **(C and D)** FITC-labeled ELANE and CX-Rhodamine-labeled Hiltonol were electroporated with exosomes in 200 µl of buffer at the settings shown on the x-axis, and ELANE and Hiltonol loading was determined based on the fluorescence standard curve. The data are presented as the mean ± SD; n = 3. **(E)** The cellular uptake of MDA-MB-231 cells or A549 cells after incubation with Texs-DiI or HELA-Exos-DiI for 2 h was evaluated by CLSM. Blue: DAPI. Red: DiI. Scale bar, 25 μm. CLSM: confocal laser scanning microscopy.

**Figure S2. Apoptosis analysis and specific cytotoxic activity of CD8^+^ T cells**

**(A)** Gating strategy for analysis of MDA-MB-231 cells; the percentages of PI-positive cells were measured. **(B)** Apoptosis analysis. Images of western blots for cleaved CASP3/CASP3 and cleaved PARP1/PARP1 in MDA-MB-231 cells. **(C)** CD8^+^ T cells were harvested from various treatment groups and incubated with target cells (MDA-MB-231, MCF7, A549, and MCF10A cells), and cytotoxicity was detected with a CCK-8 kit. The data are presented as the mean ± SD; n = 6. *t test* and one-way ANOVA were performed for statistical analysis (****: *P* < 0.0001; *: *P* < 0.05; ns: *P*> 0.05).

**Figure S3. Fluorescence imaging, pharmacokinetic curves, and safety of HELA-Exos *in vivo*.**

**(A)** *In vivo* fluorescence imaging in orthotopic MDA-MB-231 tumor-bearing mice at 2, 12, and 24 h following injection with CX-Rhodamine-labeled Hiltonol and HELA-Exos-DiI. **(B)** *In vivo* pharmacokinetic curves of Hiltonol and HELA-Exos. **(C)** Pathological examination of the major organs (the lung, heart, liver, spleen, and kidney). The tissue sections were stained with H&E. Scale bar, 100 μm. **(D)** The levels of the serum biochemical parameters ALT, AST, BUN, and CREA. The data are presented as the mean ± SD; n = 6. One-way ANOVA was performed for statistical analysis (ns: *P* > 0.05).

**Figure S4. APCs were recruited to the tumor microenvironment after HELA-Exo treatment.**

Balb/c mice with orthotopic breast cancer were sacrificed at day 30 after treatment initiation. Tumor-infiltrating DCs (CD11c^+^) and macrophages (F4/80^+^) were detected by IF. Blue: DAPI; Green: F4/80; Red: CD11c. Scale bar, 25 μm. IF: immunofluorescence.

**Figure S5. Specific cytotoxic activity of CD8^+^ T cells from the tumor microenvironment of Balb/c mice with orthotopic breast cancer**

Balb/c mice with orthotopic breast cancer were sacrificed at day 30 after treatment initiation. The tumor tissue was cut into small pieces to generate a single-cell suspension. CD8^+^ T cells were sorted and incubated with target cells (MDA-MB-231, MCF7, A549, and MCF10A cells), and cytotoxicity was detected with a CCK-8 kit. The data are presented as the mean ± SD; n = 6. A *t test* and one-way ANOVA were performed for statistical analysis (****: *P* < 0.0001; **: *P* < 0.01; ns: *P* > 0.05).

**Figure S6. Reduced systemic inflammatory toxicity of HELA-Exos.**

Balb/c mice with orthotopic breast cancer were sacrificed at day 30 after treatment initiation. The serum levels of the cytokines IL-6, IL-12, and TNF-α. The data are presented as the mean ± SD; n = 6. A *t test* was performed for statistical analysis (****: *P* < 0.0001; ns: *P*> 0.05).

**Figure S7. DC depletion efficiency in Balb/c mice and specific cytotoxic activity of CD8^+^ T cells.**

**(A)** Gating strategy for the analysis of DCs; the percentages of CD11c^+^ DCs in the lymph nodes and spleen were measured. **(B)** DC-depleted or nondepleted Balb/c mice with orthotopic breast cancer were sacrificed at Day 30 after treatment initiation. The tumor tissue was cut into small pieces to generate a single-cell suspension. CD8^+^ T cells were sorted and incubated with target cells (MDA-MB-231, MCF7, A549, and MCF10A cells), and cytotoxicity was detected with a CCK-8 kit. The data are presented as the mean ± SD; n = 6. A *t test* and one-way ANOVA were performed for statistical analysis (****: *P* < 0.0001; *: *P* < 0.05; ns: *P* > 0.05).

**Figure S8.** Tumor organoids were established from tumor tissue, and PBMCs were isolated from peripheral blood before the start of coculture. On the day of coculture, organoids were isolated from Geltrex, dissociated into single cells and plated together with PBMCs in the presence of vaccines. After 1 week of coculture, PBMCs were re-stimulated with tumor cells from organoids. After two weeks of coculture, immune infiltration in organoids was evaluated by flow cytometry and IF, and the growth-inhibitory effects in organoids of HELA-Exos were evaluated by a viability assay and live/dead cell staining.
