# Supplementary figures and images for: Engineered exosomes as an in situ DC-primed vaccine to boost antitumor immunity in breast cancer
Source: Mol Cancer. 2022 Feb 11;21:45. doi: 10.1186/s12943-022-01515-x (PMC8831689; doi:10.1186/s12943-022-01515-x)

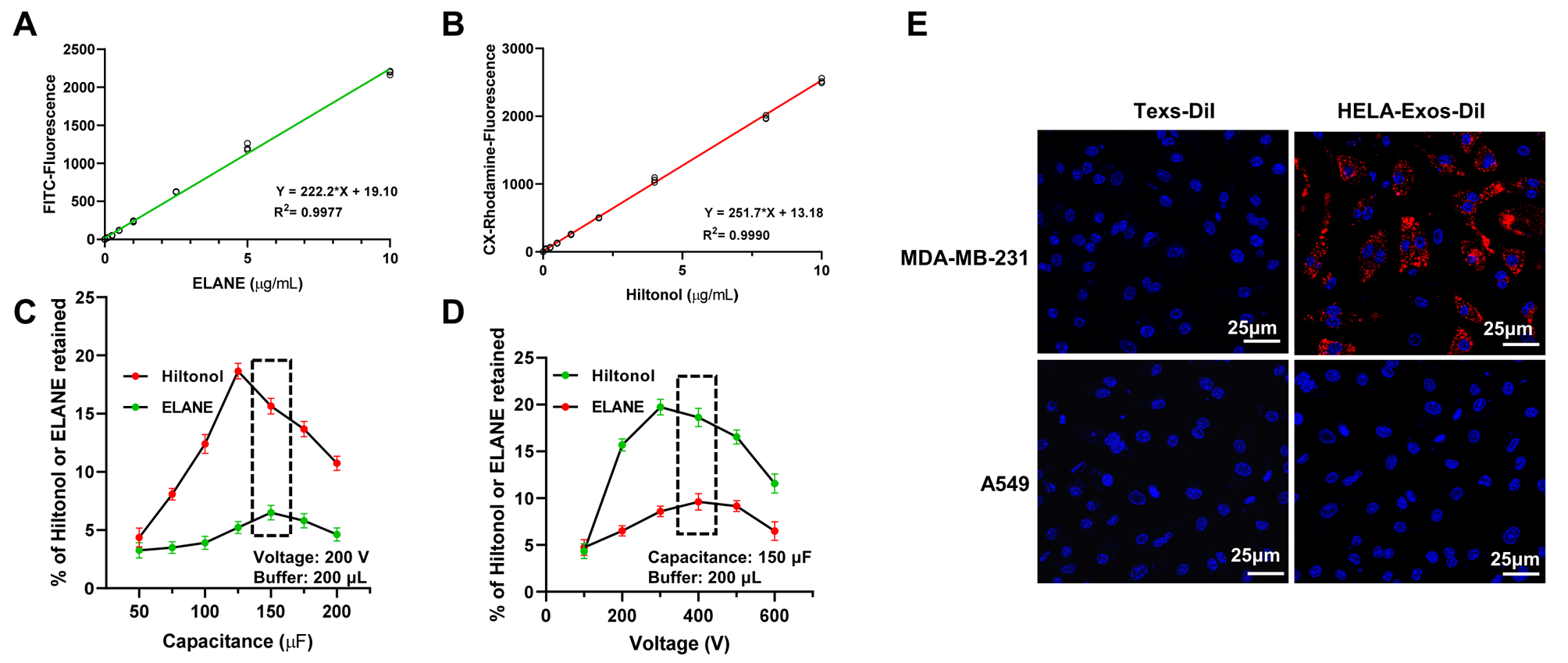

Supplement: Supplementary file 1 — Additional file 1: Figure S1. Electroporation protocol optimization and targeting of HELA-Exos. (A and B) FITC-labeled ELANE or CX-rhodamine-labeled Hiltonol dispersed in pure water at various concentrations was applied for calibration. The fluorescence standard curve of ELANE or Hiltonol was generated by measuring the fluorescence intensity at 519 nm and 597 nm. (C and D) FITC-labeled ELANE and CX-Rhodamine-labeled Hiltonol were electroporated with exosomes in 200 µl of buffer at the settings shown on the x-axis, and ELANE and Hiltonol loading was determined based on the fluorescence standard curve. The data are presented as the mean ± SD; n = 3. (E) The cellular uptake of MDA-MB-231 cells or A549 cells after incubation with Texs-DiI or HELA-Exos-DiI for 2 h was evaluated by CLSM. Blue: DAPI. Red: DiI. Scale bar, 25 μm. CLSM: confocal laser scanning microscopy. Figure S2. Apoptosis analysis and specific cytotoxic activity of CD8+ T cells. (A) Gating strategy for analysis of MDA-MB-231 cells; the percentages of PI-positive cells were measured. (B) Apoptosis analysis. Images of western blots for cleaved CASP3/CASP3 and cleaved PARP1/PARP1 in MDA-MB-231 cells. (C) CD8+ T cells were harvested from various treatment groups and incubated with target cells (MDA-MB-231, MCF7, A549, and MCF10A cells), and cytotoxicity was detected with a CCK-8 kit. The data are presented as the mean ± SD; n = 6. t test and one-way ANOVA were performed for statistical analysis (****: P < 0.0001; *: P < 0.05; ns: P > 0.05). Figure S3. Fluorescence imaging, pharmacokinetic curves, and safety of HELA-Exos in vivo. (A) In vivo fluorescence imaging in orthotopic MDA-MB-231 tumor-bearing mice at 2, 12, and 24 h following injection with CX-Rhodamine-labeled Hiltonol and HELA-Exos-DiI. (B) In vivo pharmacokinetic curves of Hiltonol and HELA-Exos. (C) Pathological examination of the major organs (the lung, heart, liver, spleen, and kidney). The tissue sections were stained with H&E. Scale bar, 100 μm [file 12943_2022_1515_MOESM1_ESM.zip › Figure S1.tif]

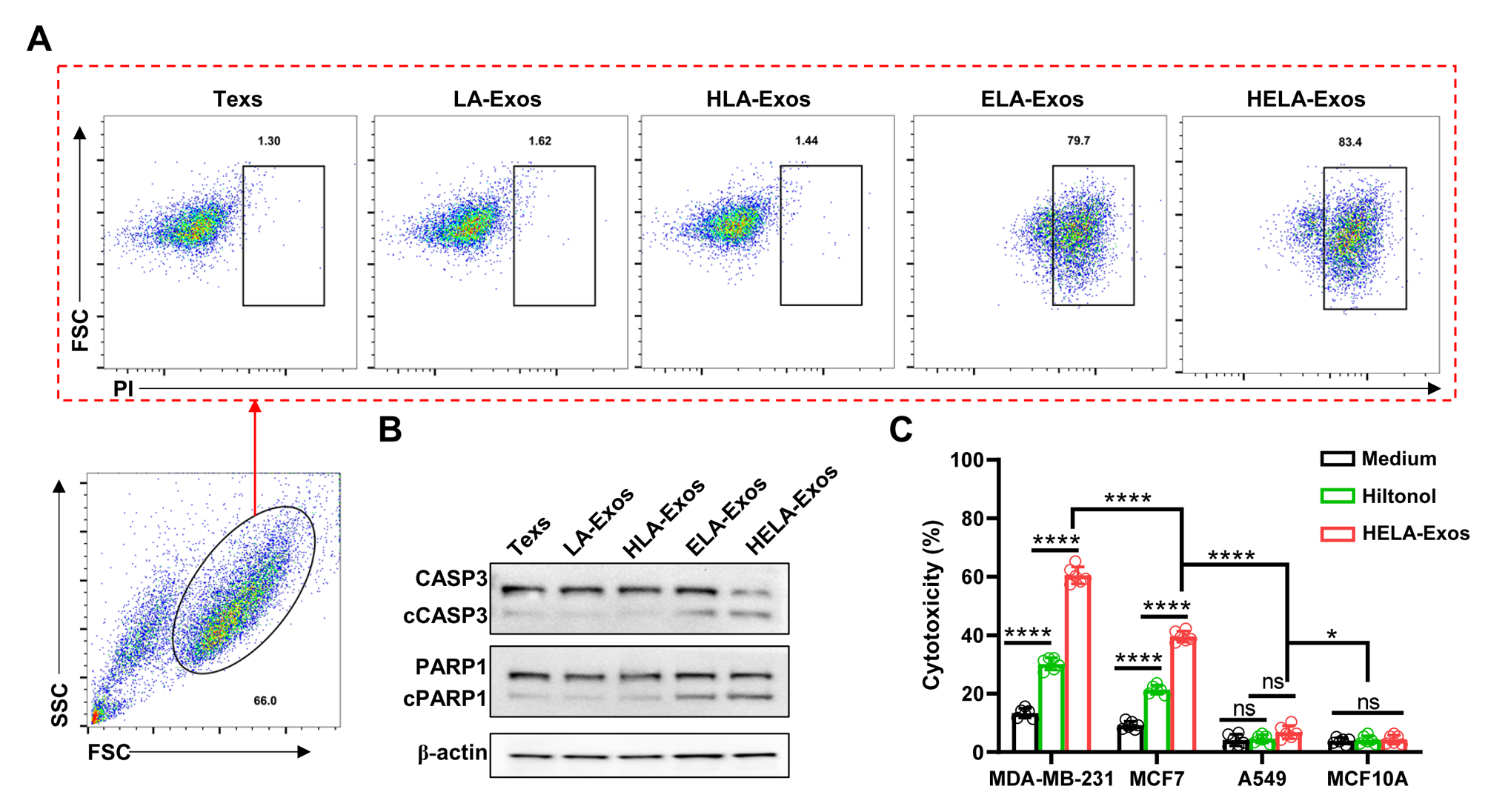

Supplement: Supplementary file 1 — Additional file 1: Figure S1. Electroporation protocol optimization and targeting of HELA-Exos. (A and B) FITC-labeled ELANE or CX-rhodamine-labeled Hiltonol dispersed in pure water at various concentrations was applied for calibration. The fluorescence standard curve of ELANE or Hiltonol was generated by measuring the fluorescence intensity at 519 nm and 597 nm. (C and D) FITC-labeled ELANE and CX-Rhodamine-labeled Hiltonol were electroporated with exosomes in 200 µl of buffer at the settings shown on the x-axis, and ELANE and Hiltonol loading was determined based on the fluorescence standard curve. The data are presented as the mean ± SD; n = 3. (E) The cellular uptake of MDA-MB-231 cells or A549 cells after incubation with Texs-DiI or HELA-Exos-DiI for 2 h was evaluated by CLSM. Blue: DAPI. Red: DiI. Scale bar, 25 μm. CLSM: confocal laser scanning microscopy. Figure S2. Apoptosis analysis and specific cytotoxic activity of CD8+ T cells. (A) Gating strategy for analysis of MDA-MB-231 cells; the percentages of PI-positive cells were measured. (B) Apoptosis analysis. Images of western blots for cleaved CASP3/CASP3 and cleaved PARP1/PARP1 in MDA-MB-231 cells. (C) CD8+ T cells were harvested from various treatment groups and incubated with target cells (MDA-MB-231, MCF7, A549, and MCF10A cells), and cytotoxicity was detected with a CCK-8 kit. The data are presented as the mean ± SD; n = 6. t test and one-way ANOVA were performed for statistical analysis (****: P < 0.0001; *: P < 0.05; ns: P > 0.05). Figure S3. Fluorescence imaging, pharmacokinetic curves, and safety of HELA-Exos in vivo. (A) In vivo fluorescence imaging in orthotopic MDA-MB-231 tumor-bearing mice at 2, 12, and 24 h following injection with CX-Rhodamine-labeled Hiltonol and HELA-Exos-DiI. (B) In vivo pharmacokinetic curves of Hiltonol and HELA-Exos. (C) Pathological examination of the major organs (the lung, heart, liver, spleen, and kidney). The tissue sections were stained with H&E. Scale bar, 100 μm [file 12943_2022_1515_MOESM1_ESM.zip › Figure S2.tif]

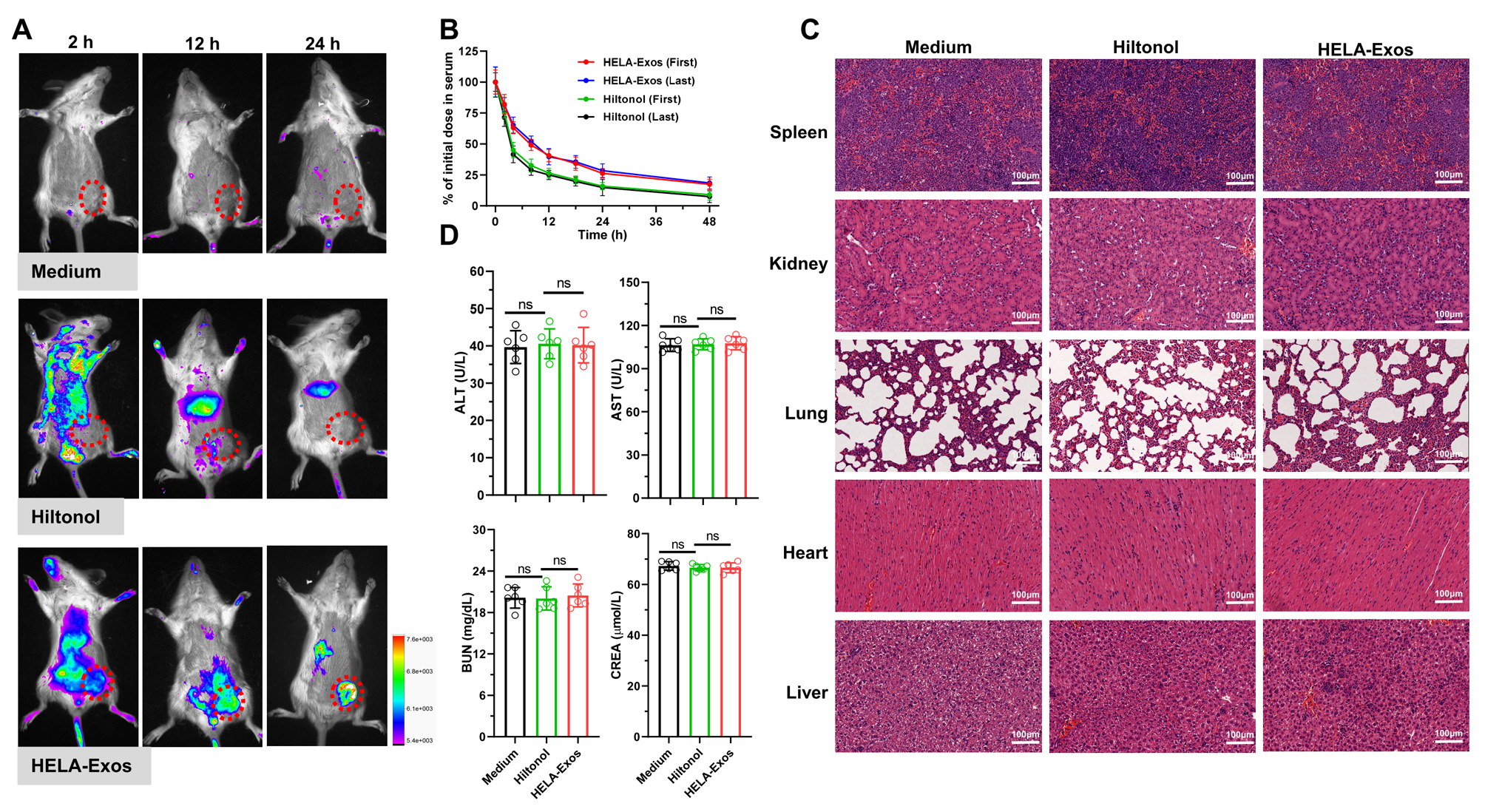

Supplement: Supplementary file 1 — Additional file 1: Figure S1. Electroporation protocol optimization and targeting of HELA-Exos. (A and B) FITC-labeled ELANE or CX-rhodamine-labeled Hiltonol dispersed in pure water at various concentrations was applied for calibration. The fluorescence standard curve of ELANE or Hiltonol was generated by measuring the fluorescence intensity at 519 nm and 597 nm. (C and D) FITC-labeled ELANE and CX-Rhodamine-labeled Hiltonol were electroporated with exosomes in 200 µl of buffer at the settings shown on the x-axis, and ELANE and Hiltonol loading was determined based on the fluorescence standard curve. The data are presented as the mean ± SD; n = 3. (E) The cellular uptake of MDA-MB-231 cells or A549 cells after incubation with Texs-DiI or HELA-Exos-DiI for 2 h was evaluated by CLSM. Blue: DAPI. Red: DiI. Scale bar, 25 μm. CLSM: confocal laser scanning microscopy. Figure S2. Apoptosis analysis and specific cytotoxic activity of CD8+ T cells. (A) Gating strategy for analysis of MDA-MB-231 cells; the percentages of PI-positive cells were measured. (B) Apoptosis analysis. Images of western blots for cleaved CASP3/CASP3 and cleaved PARP1/PARP1 in MDA-MB-231 cells. (C) CD8+ T cells were harvested from various treatment groups and incubated with target cells (MDA-MB-231, MCF7, A549, and MCF10A cells), and cytotoxicity was detected with a CCK-8 kit. The data are presented as the mean ± SD; n = 6. t test and one-way ANOVA were performed for statistical analysis (****: P < 0.0001; *: P < 0.05; ns: P > 0.05). Figure S3. Fluorescence imaging, pharmacokinetic curves, and safety of HELA-Exos in vivo. (A) In vivo fluorescence imaging in orthotopic MDA-MB-231 tumor-bearing mice at 2, 12, and 24 h following injection with CX-Rhodamine-labeled Hiltonol and HELA-Exos-DiI. (B) In vivo pharmacokinetic curves of Hiltonol and HELA-Exos. (C) Pathological examination of the major organs (the lung, heart, liver, spleen, and kidney). The tissue sections were stained with H&E. Scale bar, 100 μm [file 12943_2022_1515_MOESM1_ESM.zip › Figure S3.tif]

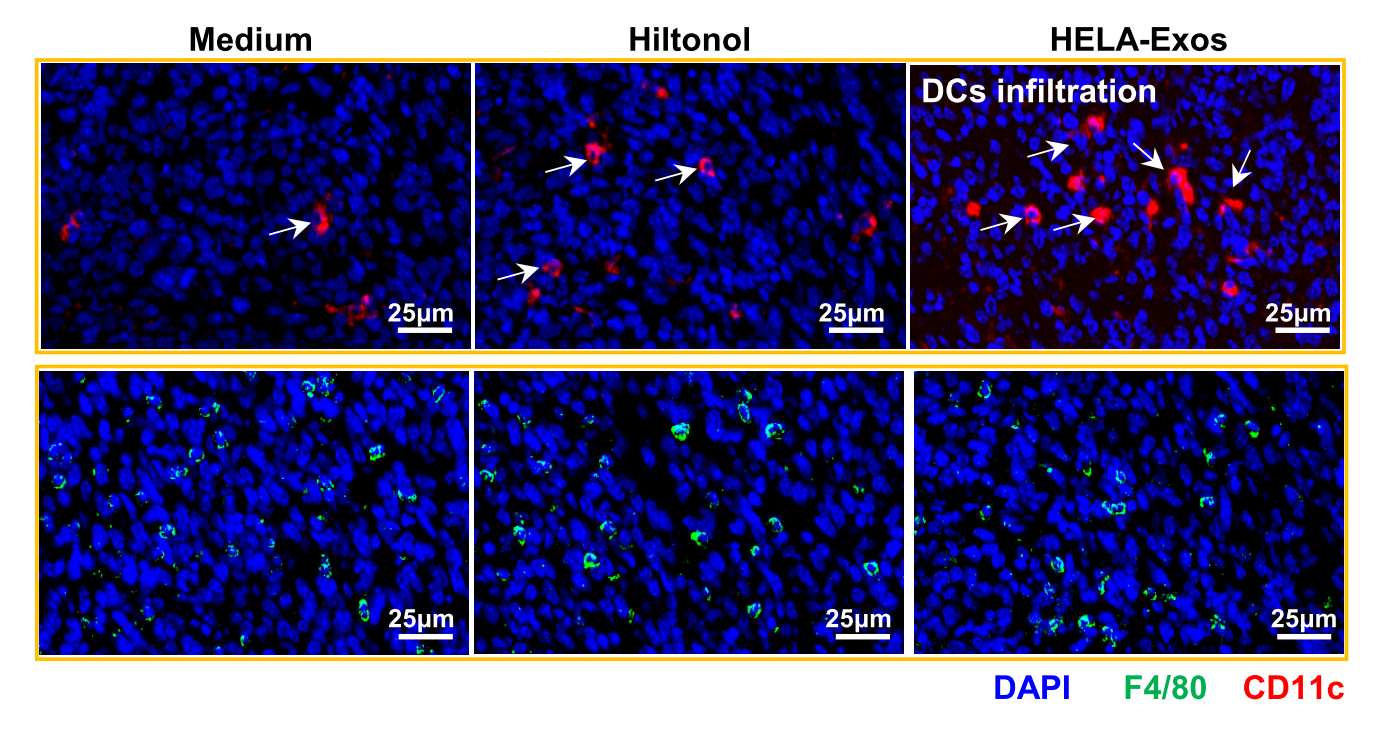

Supplement: Supplementary file 1 — Additional file 1: Figure S1. Electroporation protocol optimization and targeting of HELA-Exos. (A and B) FITC-labeled ELANE or CX-rhodamine-labeled Hiltonol dispersed in pure water at various concentrations was applied for calibration. The fluorescence standard curve of ELANE or Hiltonol was generated by measuring the fluorescence intensity at 519 nm and 597 nm. (C and D) FITC-labeled ELANE and CX-Rhodamine-labeled Hiltonol were electroporated with exosomes in 200 µl of buffer at the settings shown on the x-axis, and ELANE and Hiltonol loading was determined based on the fluorescence standard curve. The data are presented as the mean ± SD; n = 3. (E) The cellular uptake of MDA-MB-231 cells or A549 cells after incubation with Texs-DiI or HELA-Exos-DiI for 2 h was evaluated by CLSM. Blue: DAPI. Red: DiI. Scale bar, 25 μm. CLSM: confocal laser scanning microscopy. Figure S2. Apoptosis analysis and specific cytotoxic activity of CD8+ T cells. (A) Gating strategy for analysis of MDA-MB-231 cells; the percentages of PI-positive cells were measured. (B) Apoptosis analysis. Images of western blots for cleaved CASP3/CASP3 and cleaved PARP1/PARP1 in MDA-MB-231 cells. (C) CD8+ T cells were harvested from various treatment groups and incubated with target cells (MDA-MB-231, MCF7, A549, and MCF10A cells), and cytotoxicity was detected with a CCK-8 kit. The data are presented as the mean ± SD; n = 6. t test and one-way ANOVA were performed for statistical analysis (****: P < 0.0001; *: P < 0.05; ns: P > 0.05). Figure S3. Fluorescence imaging, pharmacokinetic curves, and safety of HELA-Exos in vivo. (A) In vivo fluorescence imaging in orthotopic MDA-MB-231 tumor-bearing mice at 2, 12, and 24 h following injection with CX-Rhodamine-labeled Hiltonol and HELA-Exos-DiI. (B) In vivo pharmacokinetic curves of Hiltonol and HELA-Exos. (C) Pathological examination of the major organs (the lung, heart, liver, spleen, and kidney). The tissue sections were stained with H&E. Scale bar, 100 μm [file 12943_2022_1515_MOESM1_ESM.zip › Figure S4.tif]

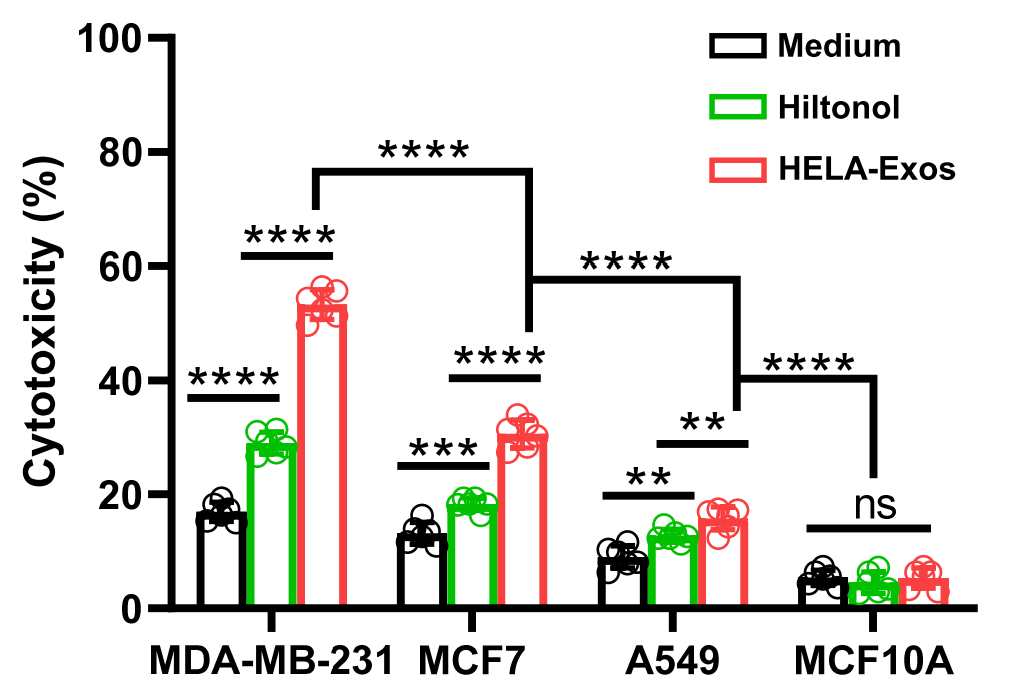

Supplement: Supplementary file 1 — Additional file 1: Figure S1. Electroporation protocol optimization and targeting of HELA-Exos. (A and B) FITC-labeled ELANE or CX-rhodamine-labeled Hiltonol dispersed in pure water at various concentrations was applied for calibration. The fluorescence standard curve of ELANE or Hiltonol was generated by measuring the fluorescence intensity at 519 nm and 597 nm. (C and D) FITC-labeled ELANE and CX-Rhodamine-labeled Hiltonol were electroporated with exosomes in 200 µl of buffer at the settings shown on the x-axis, and ELANE and Hiltonol loading was determined based on the fluorescence standard curve. The data are presented as the mean ± SD; n = 3. (E) The cellular uptake of MDA-MB-231 cells or A549 cells after incubation with Texs-DiI or HELA-Exos-DiI for 2 h was evaluated by CLSM. Blue: DAPI. Red: DiI. Scale bar, 25 μm. CLSM: confocal laser scanning microscopy. Figure S2. Apoptosis analysis and specific cytotoxic activity of CD8+ T cells. (A) Gating strategy for analysis of MDA-MB-231 cells; the percentages of PI-positive cells were measured. (B) Apoptosis analysis. Images of western blots for cleaved CASP3/CASP3 and cleaved PARP1/PARP1 in MDA-MB-231 cells. (C) CD8+ T cells were harvested from various treatment groups and incubated with target cells (MDA-MB-231, MCF7, A549, and MCF10A cells), and cytotoxicity was detected with a CCK-8 kit. The data are presented as the mean ± SD; n = 6. t test and one-way ANOVA were performed for statistical analysis (****: P < 0.0001; *: P < 0.05; ns: P > 0.05). Figure S3. Fluorescence imaging, pharmacokinetic curves, and safety of HELA-Exos in vivo. (A) In vivo fluorescence imaging in orthotopic MDA-MB-231 tumor-bearing mice at 2, 12, and 24 h following injection with CX-Rhodamine-labeled Hiltonol and HELA-Exos-DiI. (B) In vivo pharmacokinetic curves of Hiltonol and HELA-Exos. (C) Pathological examination of the major organs (the lung, heart, liver, spleen, and kidney). The tissue sections were stained with H&E. Scale bar, 100 μm [file 12943_2022_1515_MOESM1_ESM.zip › Figure S5.tif]

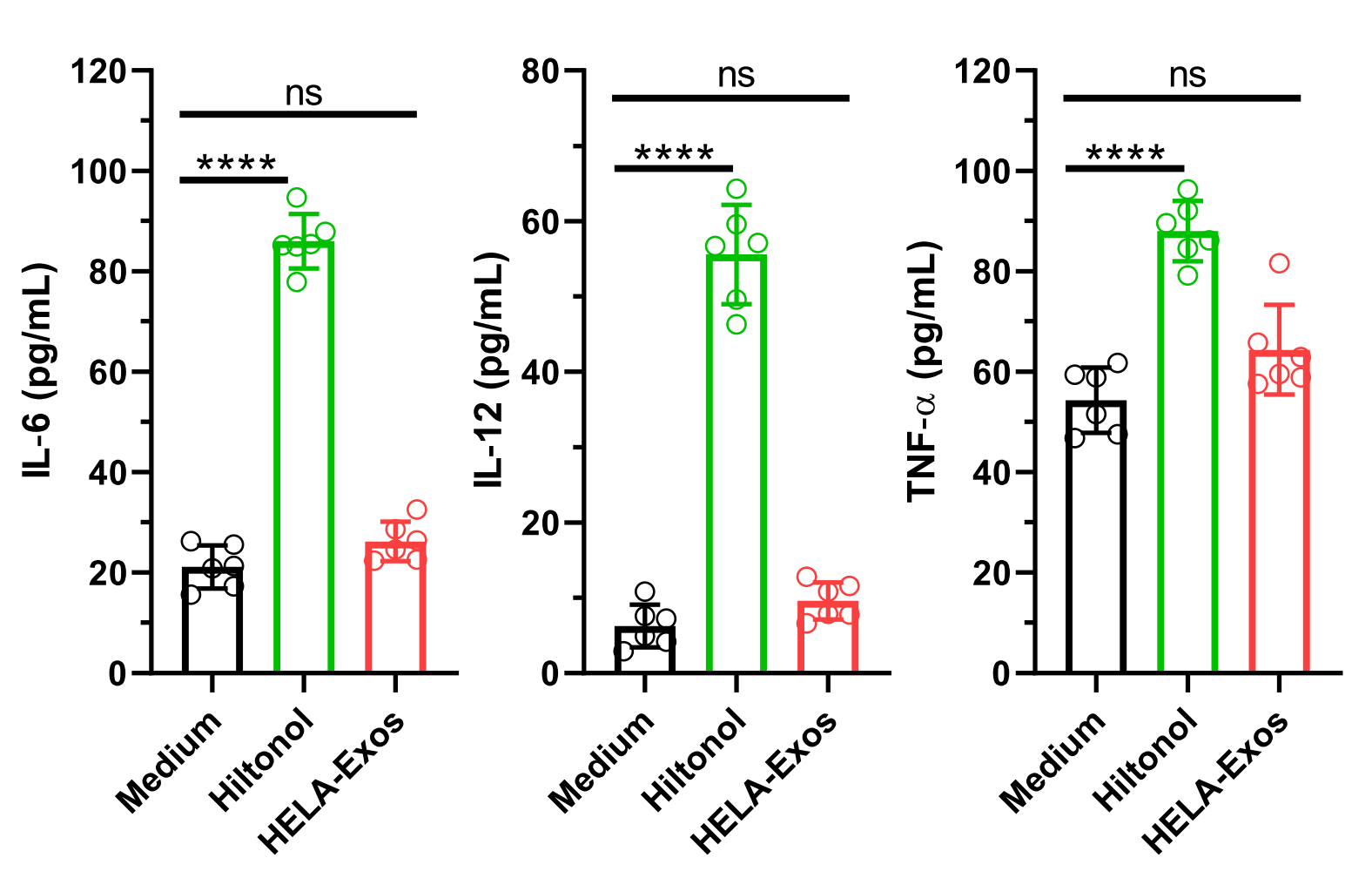

Supplement: Supplementary file 1 — Additional file 1: Figure S1. Electroporation protocol optimization and targeting of HELA-Exos. (A and B) FITC-labeled ELANE or CX-rhodamine-labeled Hiltonol dispersed in pure water at various concentrations was applied for calibration. The fluorescence standard curve of ELANE or Hiltonol was generated by measuring the fluorescence intensity at 519 nm and 597 nm. (C and D) FITC-labeled ELANE and CX-Rhodamine-labeled Hiltonol were electroporated with exosomes in 200 µl of buffer at the settings shown on the x-axis, and ELANE and Hiltonol loading was determined based on the fluorescence standard curve. The data are presented as the mean ± SD; n = 3. (E) The cellular uptake of MDA-MB-231 cells or A549 cells after incubation with Texs-DiI or HELA-Exos-DiI for 2 h was evaluated by CLSM. Blue: DAPI. Red: DiI. Scale bar, 25 μm. CLSM: confocal laser scanning microscopy. Figure S2. Apoptosis analysis and specific cytotoxic activity of CD8+ T cells. (A) Gating strategy for analysis of MDA-MB-231 cells; the percentages of PI-positive cells were measured. (B) Apoptosis analysis. Images of western blots for cleaved CASP3/CASP3 and cleaved PARP1/PARP1 in MDA-MB-231 cells. (C) CD8+ T cells were harvested from various treatment groups and incubated with target cells (MDA-MB-231, MCF7, A549, and MCF10A cells), and cytotoxicity was detected with a CCK-8 kit. The data are presented as the mean ± SD; n = 6. t test and one-way ANOVA were performed for statistical analysis (****: P < 0.0001; *: P < 0.05; ns: P > 0.05). Figure S3. Fluorescence imaging, pharmacokinetic curves, and safety of HELA-Exos in vivo. (A) In vivo fluorescence imaging in orthotopic MDA-MB-231 tumor-bearing mice at 2, 12, and 24 h following injection with CX-Rhodamine-labeled Hiltonol and HELA-Exos-DiI. (B) In vivo pharmacokinetic curves of Hiltonol and HELA-Exos. (C) Pathological examination of the major organs (the lung, heart, liver, spleen, and kidney). The tissue sections were stained with H&E. Scale bar, 100 μm [file 12943_2022_1515_MOESM1_ESM.zip › Figure S6.tif]

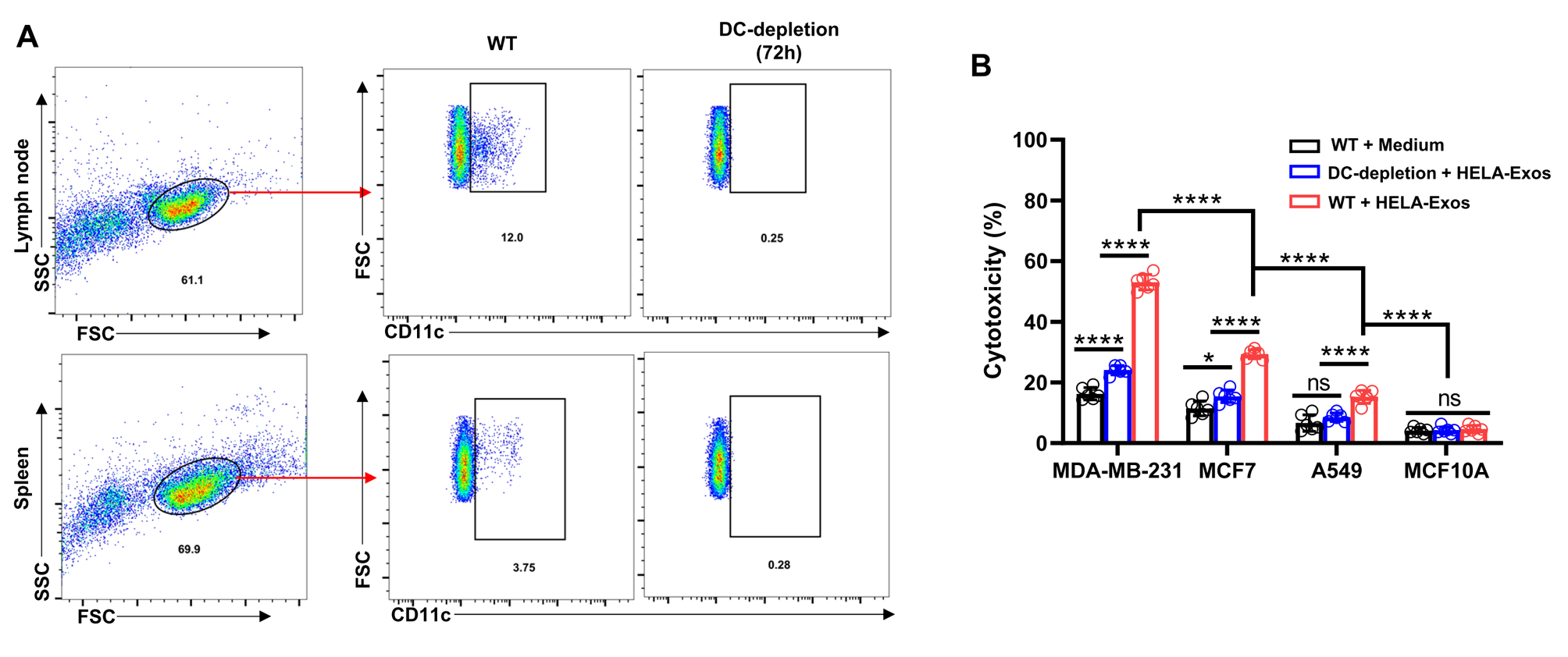

Supplement: Supplementary file 1 — Additional file 1: Figure S1. Electroporation protocol optimization and targeting of HELA-Exos. (A and B) FITC-labeled ELANE or CX-rhodamine-labeled Hiltonol dispersed in pure water at various concentrations was applied for calibration. The fluorescence standard curve of ELANE or Hiltonol was generated by measuring the fluorescence intensity at 519 nm and 597 nm. (C and D) FITC-labeled ELANE and CX-Rhodamine-labeled Hiltonol were electroporated with exosomes in 200 µl of buffer at the settings shown on the x-axis, and ELANE and Hiltonol loading was determined based on the fluorescence standard curve. The data are presented as the mean ± SD; n = 3. (E) The cellular uptake of MDA-MB-231 cells or A549 cells after incubation with Texs-DiI or HELA-Exos-DiI for 2 h was evaluated by CLSM. Blue: DAPI. Red: DiI. Scale bar, 25 μm. CLSM: confocal laser scanning microscopy. Figure S2. Apoptosis analysis and specific cytotoxic activity of CD8+ T cells. (A) Gating strategy for analysis of MDA-MB-231 cells; the percentages of PI-positive cells were measured. (B) Apoptosis analysis. Images of western blots for cleaved CASP3/CASP3 and cleaved PARP1/PARP1 in MDA-MB-231 cells. (C) CD8+ T cells were harvested from various treatment groups and incubated with target cells (MDA-MB-231, MCF7, A549, and MCF10A cells), and cytotoxicity was detected with a CCK-8 kit. The data are presented as the mean ± SD; n = 6. t test and one-way ANOVA were performed for statistical analysis (****: P < 0.0001; *: P < 0.05; ns: P > 0.05). Figure S3. Fluorescence imaging, pharmacokinetic curves, and safety of HELA-Exos in vivo. (A) In vivo fluorescence imaging in orthotopic MDA-MB-231 tumor-bearing mice at 2, 12, and 24 h following injection with CX-Rhodamine-labeled Hiltonol and HELA-Exos-DiI. (B) In vivo pharmacokinetic curves of Hiltonol and HELA-Exos. (C) Pathological examination of the major organs (the lung, heart, liver, spleen, and kidney). The tissue sections were stained with H&E. Scale bar, 100 μm [file 12943_2022_1515_MOESM1_ESM.zip › Figure S7.tif]

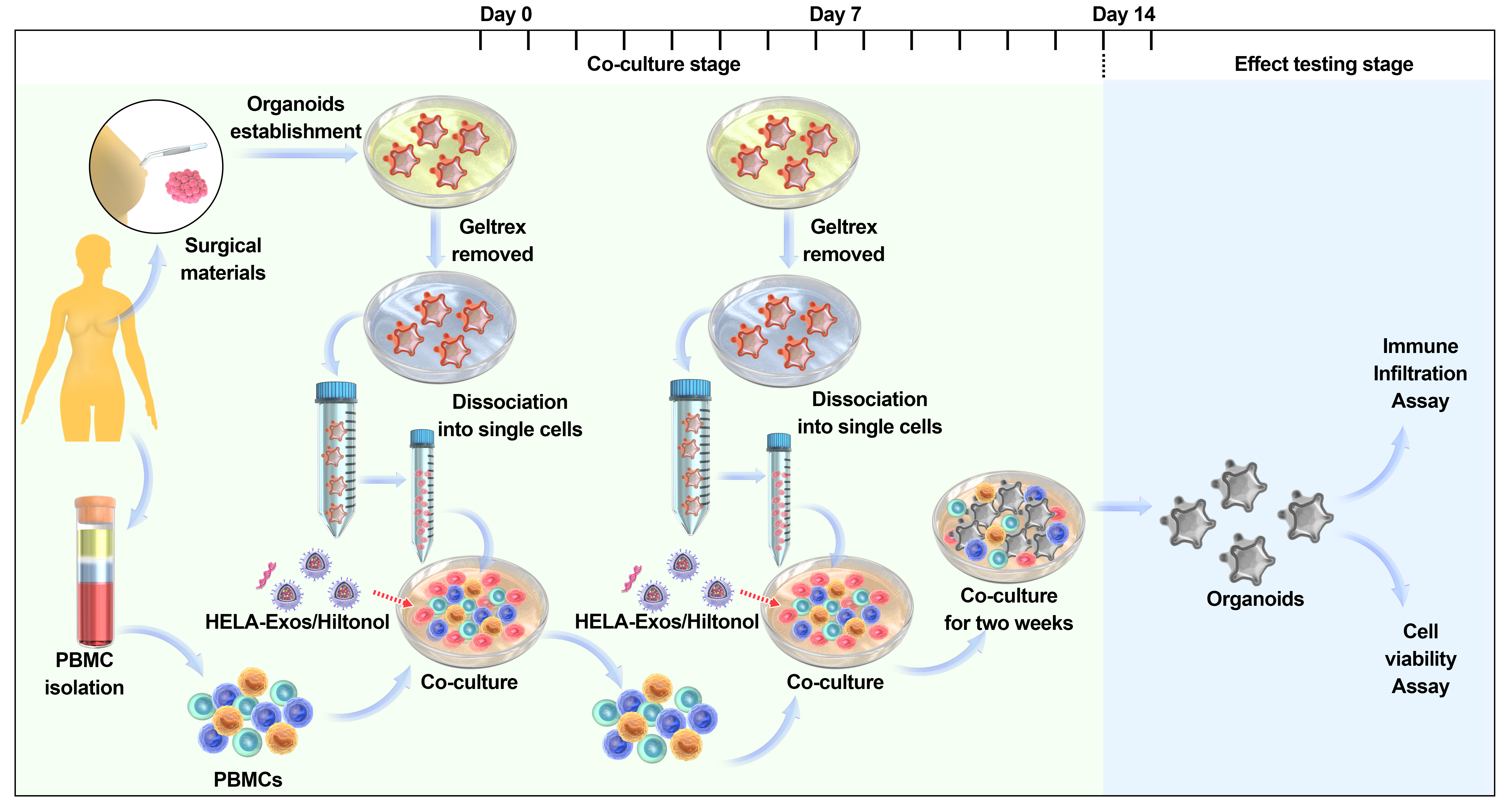

Supplement: Supplementary file 1 — Additional file 1: Figure S1. Electroporation protocol optimization and targeting of HELA-Exos. (A and B) FITC-labeled ELANE or CX-rhodamine-labeled Hiltonol dispersed in pure water at various concentrations was applied for calibration. The fluorescence standard curve of ELANE or Hiltonol was generated by measuring the fluorescence intensity at 519 nm and 597 nm. (C and D) FITC-labeled ELANE and CX-Rhodamine-labeled Hiltonol were electroporated with exosomes in 200 µl of buffer at the settings shown on the x-axis, and ELANE and Hiltonol loading was determined based on the fluorescence standard curve. The data are presented as the mean ± SD; n = 3. (E) The cellular uptake of MDA-MB-231 cells or A549 cells after incubation with Texs-DiI or HELA-Exos-DiI for 2 h was evaluated by CLSM. Blue: DAPI. Red: DiI. Scale bar, 25 μm. CLSM: confocal laser scanning microscopy. Figure S2. Apoptosis analysis and specific cytotoxic activity of CD8+ T cells. (A) Gating strategy for analysis of MDA-MB-231 cells; the percentages of PI-positive cells were measured. (B) Apoptosis analysis. Images of western blots for cleaved CASP3/CASP3 and cleaved PARP1/PARP1 in MDA-MB-231 cells. (C) CD8+ T cells were harvested from various treatment groups and incubated with target cells (MDA-MB-231, MCF7, A549, and MCF10A cells), and cytotoxicity was detected with a CCK-8 kit. The data are presented as the mean ± SD; n = 6. t test and one-way ANOVA were performed for statistical analysis (****: P < 0.0001; *: P < 0.05; ns: P > 0.05). Figure S3. Fluorescence imaging, pharmacokinetic curves, and safety of HELA-Exos in vivo. (A) In vivo fluorescence imaging in orthotopic MDA-MB-231 tumor-bearing mice at 2, 12, and 24 h following injection with CX-Rhodamine-labeled Hiltonol and HELA-Exos-DiI. (B) In vivo pharmacokinetic curves of Hiltonol and HELA-Exos. (C) Pathological examination of the major organs (the lung, heart, liver, spleen, and kidney). The tissue sections were stained with H&E. Scale bar, 100 μm [file 12943_2022_1515_MOESM1_ESM.zip › Figure S8.tif]
